# In Silico and In Vitro Studies of 4-Hydroxycoumarin-Based Heterocyclic Enamines as Potential Anti-Tumor Agents

**DOI:** 10.3390/molecules28155828

**Published:** 2023-08-02

**Authors:** Mediha Assad, Rizwan Nasir Paracha, Abu Bakar Siddique, Muhammad Ashraf Shaheen, Nadeem Ahmad, Muhammad Mustaqeem, Fariha Kanwal, Muhammad Zia Ul Mustafa, Muhammad Fayyaz ur Rehman, Sumaya Fatima, Changrui Lu

**Affiliations:** 1 College of Biological Sciences and Medical Engineering, Donghua University, 2999 North Ren Min Road, Shanghai 201620, China; 2 Department of Chemistry, Government Graduate Islamia College for Women Cantt Lahore, Lahore 54000, Pakistan; 3 Department of Chemistry, Thal University Bhakkar, Bhakkar 30000, Pakistan; 4 Institute of Chemistry, University of Sargodha, Sargodha 40100, Pakistan; abubakar.siddique@uos.edu.pk (A.B.S.);; 5 Department of Pharmacy, Comsats University Islamabad, Lahore Campus, Lahore 54000, Pakistan; 6 School of Biomedical Engineering, Shanghai Jiaotong University, Shanghai 200030, China; 7 Research Center, The Fourth Hospital of Hebei Medical University, Shijiazhuang 050011, China

**Keywords:** coumarin, hydroxycoumarin, enamines, anti-tumor

## Abstract

The present study reports the one-step synthesis of several 3-formyl-4-hydroxycouramin-derived enamines (**4a**–**4i**) in good yields (65–94%). The characterization of the synthesized compounds was carried out via advanced analytical and spectroscopic techniques, such as melting point, electron impact mass spectrometry (EI-MS), ^1^H-NMR, ^13^C-NMR, elemental analysis, FTIR, and UV-Visible spectroscopy. The reaction conditions were optimized, and the maximum yield was obtained at 3–4 h of reflux of the reactants, using 2-butanol as a solvent. The potato disc tumor assay was used to assess *Agrobacterium tumefaciens*-induced tumors to evaluate the anti-tumor activities of compounds (**4a**–**4i**), using Vinblastine as a standard drug. The compound **4g** showed the lowest IC_50_ value (1.12 ± 0.2), which is even better than standard Vinblastine (IC_50_ 7.5 ± 0.6). For further insight into their drug actions, an in silico docking of the compounds was also carried out against the CDK-8 protein. The binding energy values of compounds were found to agree with the experimental results. The compounds **4g** and **4h** showed the best affinities toward protein, with a binding energy value of −6.8 kcal/mol.

## 1. Introduction

Despite recent progress in oncology, treating tumors via chemotherapy also affects neighboring normal cells, which is still a challenge to be addressed [1]. Reports from the World Health Organization (WHO) reveal the noxious nature of cancer, which may cause more than 13 million deaths by 2030 [2]. At present, cancer is the second-leading cause of mortality (approximately 30%) in the world and will take the top position in the next few decades [3]. Therefore, the development of potent anticancer and anti-tumor agents is urgently needed.

Several drugs based on herbal formulations [4], inorganic complexes [5], and organic moieties [6] have been reported to have anti-tumor potential. These drugs can suppress or kill the abnormal cells in localized areas but have several disadvantages, such as herbal medicines having high IC_50_ values and less selectivity, while metal-based drugs are toxic to different body parts [7,8]. Metal-based drugs may also cause genetic mutations and affect renal functions [9]. In this regard, organic compounds with versatile moieties are of great interest due to their targeted actions and low IC_50_ values [10,11,12].

**Figure 1:**
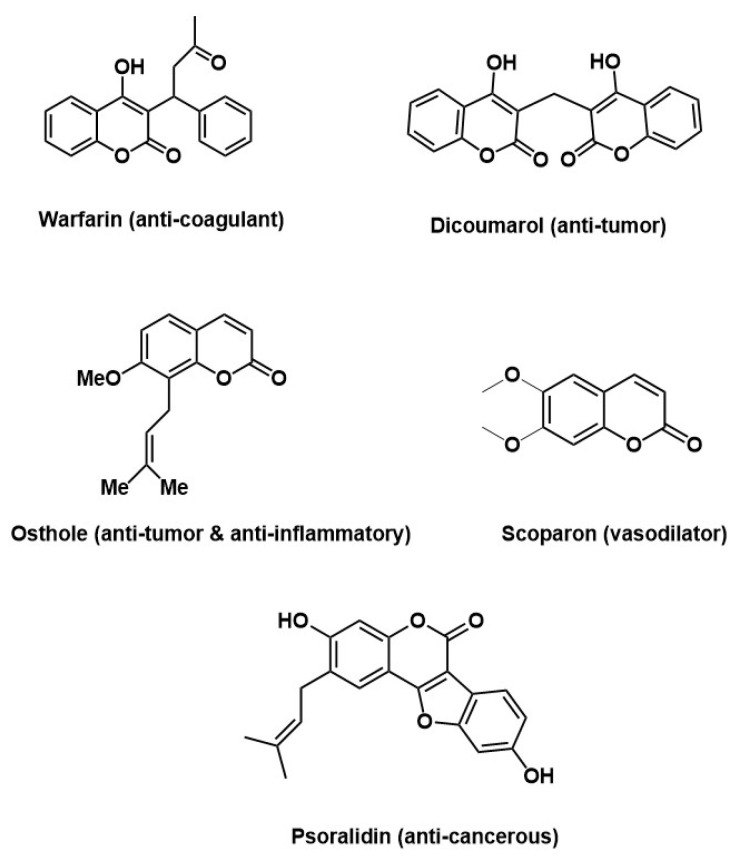
Commercially available coumarin-based drugs [17,18,19,20].

Among biologically active organic compounds, coumarin derivatives have attracted special attention from synthetic and medicinal chemists [13]. Coumarins, oxygen-bearing heterocycles with a benzopyrone skeleton, are naturally occurring compounds extracted from different plants, fungi, and bacteria [14]. This class of compounds has exhibited antimicrobial, antioxidant, antifibrotic, and anti-inflammatory effects [15] and anti-tumor potential. For example, fraxetin showed anti-tumor activity against hepatocellular carcinoma (HCC) cells [16]. Some commercially available coumarin-based drugs are shown in Figure 1 [17,18,19,20]. Among coumarin derivatives, enamines derived from coumarins are proven to be important compounds that can act as anti-tumor, antibacterial, antiproliferative, and antifungal agents [21,22]. Different strategies can be used to derivatize these heterocyclic compounds, but multistep synthesis routes often affect the yields of the final products. Therefore, one-step optimized reactions are always preferred to obtain good yields in shorter times [23].

Although many derivatives of coumarins have been tested recently, the target selection and proper delivery of anti-tumor agents to the target area still require the development of new derivatives. The properties of coumarin derivatives can be tuned by introducing different electron-withdrawing and electron-donating groups. Therefore, the development of substituted heterocyclic enamines that can selectively pass the cell barriers of tumor cells and interact with cell proteins is an interesting area [24]. Although many cell proteins can be targeted to control cell growth, the selective targeting of the CDK-8 enzyme can be a good strategy. This enzyme plays an important role in the cell cycle, i.e., gene transcription, neuronal function, insulin secretion, and glycogen synthesis, and if a drug blocks its active site, many types of tumor growth can be controlled [25,26]. Since the effects of coumarin derivatives on the CDK-8 protein have been scarcely studied, we have targeted this protein to study the in silico effects of newly synthesized coumarin-based enamines.

In the present study, the synthesis of 3-formyl-4-hydroxycoumarin-derived enamines (**4a**–**4i**) is reported with good to excellent yields (65–94%). After successful characterization, the compounds were evaluated against an *Agrobacterium tumefaciens*-mediated tumor via the potato disc tumor assay. The compounds were also tested for their in silico binding interactions with CDK-8.

## 2. Results and Discussion

### 2.1. Characterization of Compounds (***4a***–***4i***)

One pot synthesis of 3-formyl-4-hydroxycoumarin-based enamines (**4a**–**4i**) was performed by the condensation reaction of 4-hydroxy-2*H*-chromen-2-one (**1**), benzyl amine derivatives (**2**) and triethyl orthoformate (**3**) under 3–4 h reflux at 80 °C. All the compounds were characterized by spectroscopic and analytical techniques, such as FT-IR, UV-Visible spectroscopy, ^1^H-NMR, ^13^C-NMR, EI-MS, elemental analysis, and melting point. In FTIR spectra, the synthesis of target compounds was indicated by the absorption bands in the range of 3200–3400, 1680, and 1600 cm^−1^, attributed to the vibrations of NH, C=O, and C=N functional groups, respectively. The appearance of the imine group peak (C=N) at 1600 cm^−1^ was the main indication of product formation. In UV-Vis spectra, electronic transitions in the range of 19,380–19,500 cm^−1^ were assigned to the π→π* and n→π* transitions in compounds (**4a**–**4i**). High Molar absorptivity constant values (Є = 12,350 L·mol^−1^·cm^−1^) were observed for π→π* transitions at lower wavelength than n→π* transitions (Є= 945 L·mol^−1^ cm^−1^) occurring at longer wavelength region. These transitions were in accordance with the color of purified compounds.

**Figure 2:**
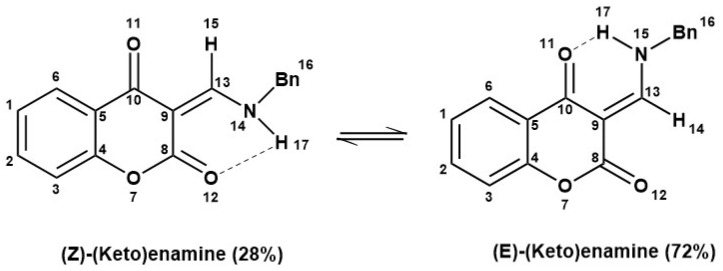
Tautomerism of (E)-(Keto)enamine and (Z)-(Keto)enamine.

The ^1^H-NMR spectra (Figures S1A–S9A) revealed the presence of both (Z) and (E) isomers, as shown in Figure 2. The β-hydrogen *cis-* to the cyclic ester functional group of an α,β-unsaturated ester appeared up field, hence representing the (E) isomer of hydroxy-enamine, while the exocyclic vinyl proton in (Z) isomers appeared at a higher chemical shift (δ) value. In the ^1^H-NMR spectra, the signals at the lowest chemical shift (3.82–3.94 ppm) were assigned to the methoxy protons, and two signals around 4.68 and 4.72 ppm represented the methylene protons in (E) and (Z) isomers, respectively. The aromatic protons of both phenyl rings appeared in the range of 6.83–7.85 ppm. Finally, two broad signals around 10–12 ppm were assigned to NH protons of both (Z) and (E) isomers. The signal at a higher chemical shift value was assigned to the (E) isomer because of strong intramolecular H-bonding between H(17) and O(11), while the signal at the lower chemical shift value was assigned to the (Z) isomer due to weak H-bonding between O(12) and H(17) [27,28]. The intensity of the (15) signal around 8.5–8.8 ppm due to (E) and (Z) isomers indicated the presence of the (E) isomer in a higher ratio (72%) than the (Z) isomer (28%), and this was attributed to the extra-stability of (E) isomer due to strong H-bonding.

The ^13^C-NMR spectra (Figures S1B–S9B) decoupled with (^13^C-^1^H/^13^C-^19^F) signals showed the chemical shift value for each carbon atom of the designed compound. The coupling constant values of (^13^C-^1^H/^13^C-^19^F) coupling are very high, in the range of 200–300 Hz. Therefore, the decoupling of (^13^C-^1^H/^13^C-^19^F) signals was carried out to avoid the peak broadening and accurate determination of chemical shift values. The signals at chemical shift values of 50–55 ppm, 110–150 ppm, and 160–165 ppm were assigned to the carbons of methylene, aromatic ring, and carbonyl groups, respectively. Elemental analysis of the compounds was carried out to check the elemental composition and purity. The percentages of elements (C, H, and N) were found in agreement with the calculated values, indicating the purity and the formation of desired compounds. Moreover, *m*/*z* value molecular ion peaks in the EI-MS spectrum were also found in accordance with the predicted molecular mass of compounds.

### 2.2. Optimization of Reaction Conditions

Several catalysts, including acids, bases, and metal salts, were screened to observe their effect on reaction yield and time, as shown in Table 1. Among these solvents, 2-butanol was the best choice with respect to the product yield. Neutral conditions of the reaction were found to be the most suitable for the product yield. Moreover, no effect was observed on the isomeric forms of the compound. Both isomers (E and Z forms) were found in the reaction mixture, with and without a catalyst.

### 2.3. Anti-Tumor Activity

The anti-tumor activity of compounds (**4a**–**4i**) was performed following the potato disc tumor assay [29]. Potato disc tumor assay is a rapid, reliable, and preliminary method to evaluate the anti-tumor potential of compounds in laboratories. This assay is used to evaluate the ant-mitotic potential of compounds to control tumor growth. *A. tumefaciens-induced* tumor is a common disease in plants [30]. These bacteria contain tumor-inducing plasmids, which cause uncontrolled cell growth in plants, and large sized tumors appear on their surfaces. The histology and nucleic acid have shown the close similarity of these tumors to human cancer cells. Therefore, this test was performed to evaluate the anti-tumor potential of compounds [31,32].

**Figure 3:**
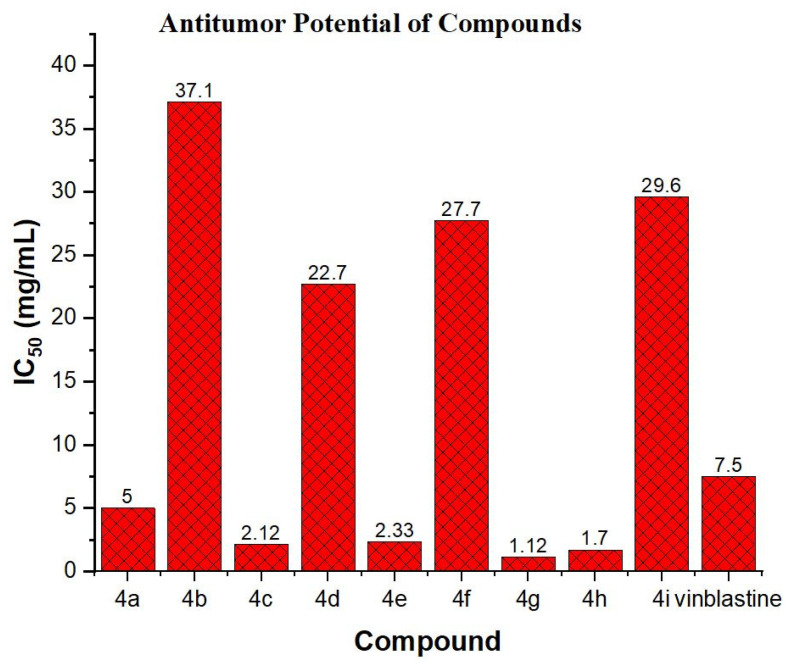
IC_50_ values of compounds (**4a**–**4i**) against *Agrobacterium tumefaciens-*induced tumor in potato discs.

Interestingly, it was observed that compounds (**4a**, **4c**, **4e**, **4g**, and **4h**) have better anti-tumor activities than the standard drug Vinblastine. Of these compounds, **4g** showed the strongest cytotoxic activity with IC_50_ = 1.12 ± 0.02 mg/mL, while other compounds **4b**, **4d**, **4f**, and **4i** were observed to show moderate potency with IC_50_ values between 22.7–37.1 mg/mL, as shown in Table S1. A comparison of the IC50 values of compounds has been depicted in Figure 3.

### 2.4. Molecular Docking Studies

Probing in silico interactions of synthetic drug candidates with the targeted proteins or biomolecules is an effective strategy to evaluate their efficacy and mode of action [33,34]. Cyclin dependent kinases (CDKs) are actively involved in the cell cycle and other cellular phenomena, including gene transcription, neuronal function, insulin secretion, and glycogen synthesis. CDKs inhibitors are extensively studied in cancer therapy because these agents can block the cell cycle and control cell proliferation by inhibiting the CDK enzyme activity [35]. CDK-8 is an important enzyme that plays the main role in the cell cycle; therefore, this enzyme is targeted for the in silico studies of compounds [36]. 

**Figure 4:**
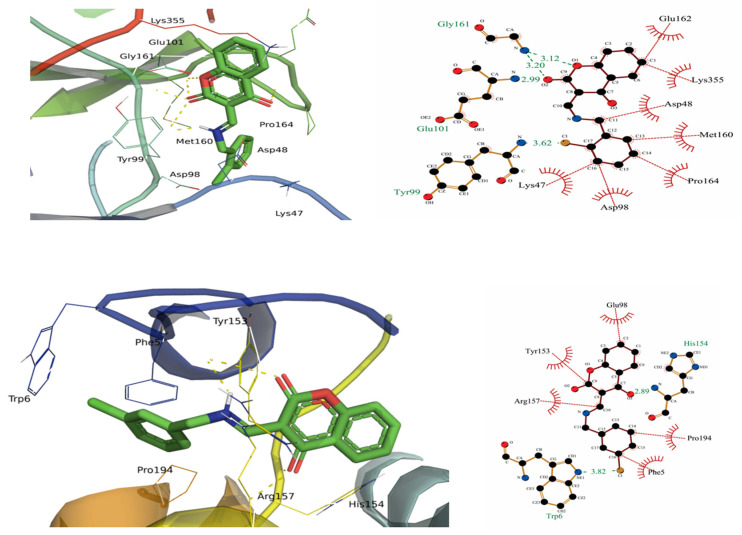
Docking simulation of the interaction between compounds (**4g** and **4h**) and CDK-8.

Docking interactions with CDK-8 revealed the good binding energies of all the compounds. The docking score of each compound with CDK-8 is given in Table 2. Among all the compounds, **4g** and **4h** exhibited the best binding score, consistent with the experimental studies (Figure 4). Both types of interactions (H-bonding and hydrophobic interactions) were observed between compounds and CDK-8. Amino groups of Tyr-99, Glu-101, His-154, Gly-161, and Phe-195 were involved in the H-bonding with the oxygen atoms of ligands, while hydrophobic interactions were observed between Phe-5, Lys-47, Asp-98, Tyr-153, and Tyr-156 residues and carbon atoms of ligands. In addition to synthesized compounds, the docking simulations of vinblastine and native protein-bound ligand, i.e., 8-(3-(3-amino-1*H*-indazol-6-yl)-5-chloropyridin-4-yl)-2,8-diazaspiro[4.5]decan-1-one (5XG) were performed with the CDK-8. Vinblastine and 5XG exhibited good binding scores of −9.11 and −9.18 kcal/mol, respectively. However, **4a**, **4c**, **4e**, **4g**, and **4h** bind better with CDK-8 than vinblastine and native ligand, 5XG. These interactions showed that the protein-ligand complexes are highly stable. Docking simulations of compounds (**4a**, **4b**, **4c**, **4d**, **4e**, **4f**, **4i**, and vinblastine) are presented in Figure S10.

## 3. Experimental Section

### 3.1. Materials and Methods

Analytical grade chemicals purchased from Merck and Sigma Aldrich (Darmstadt, Germany) were used without further purification. Solvents were used in raw form for a solubility check. Melting points were determined using the Fisher-Johns melting point apparatus (Thermo Scientific, Waltham, MA, USA). Spectrophotometer (SHIMADZU UV 240, Kyoto, Japan) and FT-IR (Shimadzu Prestige-21, Kyoto, Japan) were used to record UV/Visible and FTIR spectra, respectively. Nuclear magnetic resonance spectra were recorded in DMSO-d_6_ on Bruker AM 300 spectrometer (Rhenistetten-Forchheim, Germany) operating at 300 MHz and using TMS as an internal standard. The chemical shifts (δ) are reported in parts per million (ppm) and coupling constants in Hz.

### 3.2. Synthesis of Substituted 3-((Benzylamino)methylene)-3H-chromene-2,4-dione (***4a***–***4i***)

**Figure 5:**
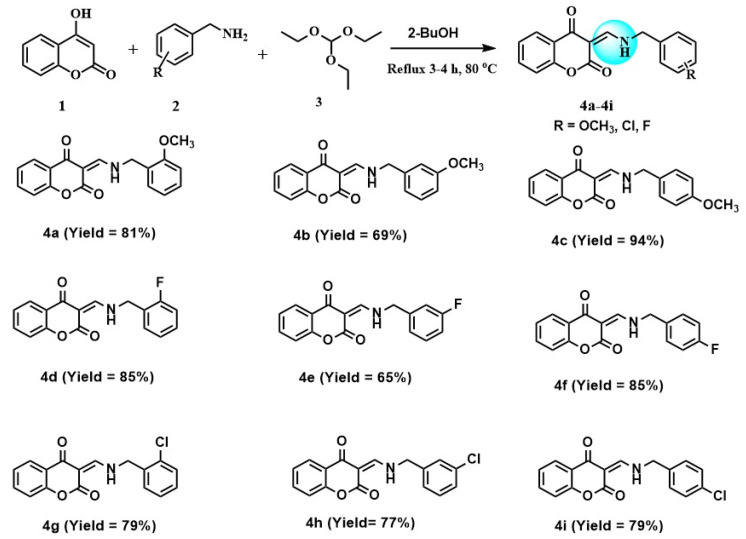
General scheme of synthesis and structures of 3-formyl-4-hydroxycoumarin-based enamines (**4a**–**4i**).

Synthesis of substituted *3-((benzylamino)methylene)-3H-chromene-2,4-dione* is reported following the literature [37,38,39] with slight modification. A total of 2.5 mmol of substituted benzylamine (**2**) in 2-butanol was added dropwise in an equimolar solution of 4-hydroxycoumarin (**1**) in the same solvent, with a slight excess of ethyl orthoformate under reflux conditions. The progress of the reaction was continuously monitored by TLC. After 3–4 h reflux, the product precipitates adhered to the flask surfaces, indicating the completion of the reaction. Precipitates were filtered, washed with ethanol, dried in an oven, and stored in airtight bottles as a purified product. Figure 5 represents the general scheme of synthesis and structures of compounds (**4a**–**4i**).

*3-((2-methoxybenzylamino)methylene)-3H-chromene-2,4-dione (* **4a** *)*

Yield: 81%; pink crystalline solid; FTIR (KBr, cm^−1^**)**: 3380 (N-H), 1690 (C=O), 1626 (C=C); λ_max_ (cm^−1^): 19,495; ^1^H-NMR: (300 MHz, DMSO-d_6_) δ (ppm): 3.94 (s, OCH_3_) 4.66 (d, *J* = 6.0 Hz, 2H, CH_2_ (E)); 4.70 (d, *J* = 6.0 Hz, 2H, CH_2_ (Z)), 6.95 (dd, *J* = 9.0 Hz & *J* = 6.0 Hz, 2H, Ph, Ar-H & Ar-H), 7.00–7.28(m, 2H, Ar-H), 7.34–7.40 (m, 2H, Ar-H), 7.53–7.56 (m, 1H, Ar-H), 8.00 (dd *J*_5,6_ = 6.0 Hz, *J*_5,7_ = 3.0 Hz, 1H, Ar-H (E)), 8.02 (dd *J*_5,6_ = 6.0 Hz, *J*_5,7_ = 3.0 Hz, 1H, Ar-H(Z)), 8.52 (d, *J*_H(9),NH_ = 12 Hz, C_9_-H (E)), 8.65 (d, *J*_H(9),NH_ = 12 Hz, C_9_-H(Z)); 10.62 (S, br, 1H, NH (Z)), 12.07 (s, br, 1H, NH, (E) (Figure S1A); ^13^C-NMR (300 MHz, CDCl_3_) 51.21, 55.54, 110.86, 117.24, 120.92, 122.90, 123.85, 124.04, 125.64, 126.35, 129.68, 130.55, 134.12, 157.58, 160.50, 162.14 (Figure S1B); EI-MS, *m*/*z* (%): 309(M^+^,100), 294(7.1), 278(3.6), 249(1.5), 188(12.5), 176(7.1), 147(1.8), 134(5.6), 121(64.8), 91(59.8), 82.9(26.8), 65(7.1), 51(2.4), 44(19.0); elemental analysis: C_18_H_15_NO_4_: (309.32); calculated (%): C; 69.89, H; 4.89, N; 4.53; found (%): C; 69.90, H; 4.85, N; 4.53; melting point: 218 °C.

*3-((3-methoxybenzylamino)methylene)-3H-chromene-2,4-dione (* **4b** *)*

Yield: 69%; Pink amorphous solid; FTIR (KBr, cm^−1^): 3400 (N-H), 1701 (C=O), 1647 (C=C); λ_max_ (cm^−1^): 19,435; ^1^H-NMR: (300 MHz, DMSO-d_6_) δ (ppm): 3.82 (s, OCH_3_), 4.68 (d, *J* = 6.0 Hz, 2H, CH_2_ (E)), 4.72 (d, *J* = 6.0 Hz, 2H, CH_2_ (Z)), 6.83 (t, *J* = 3.0 Hz, 1H, ph, C_2_′-H), 6.88–6.93 (m, 2H, Ar-H), 7.24–7.33 (m, 1H, Ar-H), 8.00 (dd *J*_5,6_ = 6.0 Hz, *J*_5,7_ = 3.0 Hz, 1H, C_5_-H (E)), 8.02 (dd *J*_5,6_ = 6.0 Hz, *J*_5,7_ = 3.0 Hz, 1H, Ar-H (Z)), 8.50 (d, *J*_H(9),NH_ = 15 Hz, C_9_-H (E)), 8.66 (d, *J*_H(9),NH_ = 15 Hz, C_9_-H (Z)), 10.48 (s Br, 1H, NH (Z)), 12.14 (s, br, 1H, NH (E)) (Figure S2A); ^13^C-NMR (300 MHz, CDCl_3_); 55.44, 54.57, 97.35, 113.53, 114.13, 117.32, 119.87, 120.53, 123.99, 124.20, 130.44, 134.39, 135.95, 160.27, 160.68, 162.24 (Figure S2B); EI-MS, *m*/*z* (%): 309(M^+^,100), 294(12), 292(22.9), 279(5.1), 249(3.2), 188(29.7), 176(8.8), 172(65.9), 160(12.5), 147(8.7), 134(12.7), 121(58.9), 91(18), 65(6.4), 51(2.7), 44(9.2).; elemental analysis: C_18_H_15_NO_4_: (309.32); calculated (%): C; 69.89, H; 4.89, N; 4.53; found (%): C; 69.90, H; 4.85, N; 4.53; melting point: 118 °C.

*3-((4-methoxybenzylamino)methylene)-3H-chromene-2,4-dione (* **4c** *)*

Yield: 94%; Pink amorphous solid; FTIR (KBr, cm^−1^**)**: 3400 (N-H), 1705 (C=O), 1607 (C=C); λ_max_ (cm^−1^): 19,460; ^1^H-NMR: (300 MHz, DMSO-d_6_) δ (ppm): 3.83 (s, OCH_3_), 4.65 (d, *J* =6.0 Hz, 2H, CH_2_ (E)), 4.68(d, *J* = 6.0 Hz, 2H, CH_2_ (Z)), 6.93 (d, *J* = 3.0 Hz, 2H, Ar-H), 7.23–7.28(m, 4 H, Ar-H), 7.55–7.58 (m, 1H, Ar-H), 8.00 (dd *J*_5,6_ = 6.0 Hz, *J*_5,7_ = 3.0 Hz, 1H, Ar-H (E)), 8.02 (dd *J*_5,6_ = 6.0 Hz, *J*_5,7_ = 3.0 Hz, 1H, Ar-H(Z)), 8.48 (d, *J*_H(9),NH_ = 15 Hz, C_9_-H (E)), 8.64 (d, *J*_H(9),NH_ = 15 Hz, C_9_-H(Z)), 10.44 (s, br, 1H, NH (Z)), 12.11 (s, br,1H, NH, (E)) (Figure S3A); ^13^C-NMR (300 MHz, CDCl_3_): 54.05, 54.14, 55.38, 99.99, 114.72, 117.30, 123.97, 124.17, 125.68, 126.29, 126.40, 129.32, 134.33, 160.29, 161.86 (Figure S3B); EI-MS, *m*/*z* (%): 309(89.5), 292(7.1), 280(1.5), 188(5.7), 176(4.0), 172(7.5), 160(2.8), 147(1.6), 134(5.2), 121(M^+^ 100), 91(3.7), 63(2.5), 51(1.7), 44(14.3); elemental analysis: C_18_H_15_NO_4_: (309.32); calculated (%): C; 69.89, H; 4.89, N; 4.53; found (%): C; 69.90, H; 4.85, N; 4.53; melting point: 182 °C).

*3-((2-fluorobenzylamino)methylene)-3H-chromene-2,4-dione (* **4d** *)*

Yield: 85%; Light pink amorphous solid; FTIR (KBr, cm^−1^**)**: 3220 (N-H), 1713 (C=O), 1634 (C=C); λ_max_ (cm^−1^): 19,390; ^1^H-NMR: (300 MHz, DMSO-d_6_) δ (ppm): 4.75 (d, *J* = 6.0 Hz, 2H, CH_2_ (E), 4.79 (d, *J* = 6.0 Hz, 2H, CH_2_ (Z), 7.15–7.39 (m, 2H, Ar-H), 7.55–7.58 (m, 4 H, Ar-H), 7.55–7.58 (m, 1H, Ar-H), 8.01 (dd *J*_5,6_ = 6.0, *J*_5,7_ = 3.0 Hz, 1H, Ar-H (E)), 8.02 (dd *J*_5,6_ = 6.0, *J*_5,7_ = 3.0 Hz, 1H, C_5_-H(Z)), 8.53 (d, *J*_H(9), NH_ = 15 Hz, C_9_-H (E)), 8.68 (d, *J*_H(9), NH_ = 15 Hz, C_9_-H(Z)), 10.46 (s, br, 1H, NH (Z)), 12.15 (s, br, 1H, NH (E)) (Figure S4A); ^13^C-NMR (300 MHz, CDCl_3_): 48.86, 48.91, 115.98, 116.26, 117.32, 124.00, 124.88, 124.93, 125.71, 126.44, 129.83, 129.88, 131.00, 131.11, 134.44, 160.84, 162.43 (Figure S4B); EI-MS, *m*/*z* (%): 297(M^+^ 100), 280(10.7), 268(2.4), 252(1.8), 203(3.3), 188(87.5), 176(25.6), 160(16.2), 148.1(10.2), 135(2.2), 121(36.4), 109(94.3), 92(5.6), 82.9(19.7), 63(7.5), 51(2.9), 44(9.5); elemental analysis: C_17_H_12_NO_3_F: (297.30); calculated (%): C; 68.68, H; 4.07, N; 4.71; found (%): C; 68.69, H; 4.04, N; 4.71; melting point: 140 °C.

*3-((3-fluorobenzylamino)methylene)-3H-chromene-2,4-dione **(*** **4e** ***)***

Yield: 65%, Light pink crystalline solid; FTIR (KBr, cm^−1^): 3215 (N-H), 1701 (C=O), 1632 (C=C); λ_max_ (cm^−1^): 19,480; ^1^H-NMR: (300 MHz, DMSO-d_6_) δ (ppm): 4.71(d, *J* = 6.0 Hz, 2H, CH_2_ (E), 4.75 (d, *J* = 6.0 Hz, 2H, CH_2_ (Z)), 7.04 (d, *J* = 9.0 Hz, 2H, Ar-H), 7.07 (d, *J* = 6.0 Hz, 1H, Ar-H),7.09–7.41(m, 2H, Ar-H), 7.56–7.59 (m, 1H, Ar-H), 7.59–7.61 (m, 1H, Ar-H), 8.01 (dd *J*_5,6_ = 6.0 Hz, *J*_5,7_ = 3.0 Hz, 1H, C_5_-H (E)), 8.02 (dd *J*_5,6_ = 6.0 Hz, *J*_5,7_ = 3.0 Hz, 1H, Ar-H (Z)), 8.53 (d, *J*_H(9),NH_ = 15 Hz, C_9_-H (E)), 8.68 (d, *J*_H(9),NH_ = 15 Hz, C_9_-H (Z)), 10.50 (s, br, 1H, NH (Z)), 12.16 (s, br, 1H, NH (E)) (Figure S5A); ^13^C-NMR (300 MHz, CDCl_3_): 53.83, 53.96, 97.56, 114.57, 114.86, 115.74, 116.02, 117.30, 117.35, 120.45, 123.18, 123.22, 124.06, 124.27, 125.73, 129.83, 125.73, 126.43, 130.96, 131.07, 134.53, 136.98 154.93, 160.85, 162.44 (Figure S5B); EI-MS, *m*/*z* (%): 293(M^+^ 100), 280(20.8), 268(3.2), 252(3.0), 203(2.9), 188(67.1), 176(24.3), 160(41), 148.1(12.7), 135(4.9), 121(39.9), 109(63.5), 92(5.9), 82.9(44.2), 63(5.5), 51(2.7), 44(8.4); elemental analysis: C_17_H_12_NO_3_F: (297.30); calculated (%): C; 68.68, H; 4.07, N; 4.71; found (%): C; 68.69, H; 4.04, N; 4.71; melting point: 180 °C.

*3-((4-fluorobenzylamino)methylene)-3H-chromene-2,4-dione* (**4f**)

Yield: 85%; Light pink crystalline solid; FTIR (KBr, cm^−1^): 3050 (N-H), 1715 (C=O), 1638 (C=C); λ_max_ (cm^−1^): 19,450; ^1^H-NMR: (300 MHz, DMSO-d_6_) δ (ppm): 4.71 (d, *J* = 6.0 Hz, 2H, CH_2_ (E)), 4.75 (d, *J* = 6.0 Hz, 2H, CH_2_ (Z); 7.09 (d, *J* = 9.0 Hz 2H, Ar-H), 7.33–7.56 (m, 4H, Ar-H), 7.56–7.58 (m, 1Н,Ar-H), 8.01 (dd *J*_5,6_ = 6.0 Hz, *J*_5,7_ = 3.0 Hz, 1H, Ar-H(E)), 8.02 (dd *J*_5,6_ = 6.0 Hz, *J*_5,7_ = 3.0 Hz, 1H, Ar-H(Z)), 8.50 (d, *J*_H(9),NH_ = 15 Hz, C_9_-H(E)), 8.71 (d, *J*_H(9),NH_ = 15 Hz, C_9_-H (Z)), 10.46 (s, br, 1H, NH (Z)), 12.13 (s, br, 1H, NH, (E)) (Figure S6A); ^13^C-NMR (300 MHz, CDCl_3_): 53.77, 53.89, 97.42, 116.21, 116.50, 116.70, 117.34, 124.05, 124.45, 124.81, 125.71, 126.43, 129.57, 132.56, 134.49, 160.58, 162.16, 164.56. EI-MS, *m*/*z* (%): 297(M^+^ 100), 280(11.3), 268(2.0), 252(1.4), 203(1.6), 188(34), 176(15.3), 160(17.7), 148.1(7.1), 135(2.2), 121(21.4), 109(92.2), 92(3.4), 63(4.9), 51(1.8), 44(16.5) (Figure S6B); elemental analysis: C_17_H_12_NO_3_F: (297.30); calculated (%): C; 68.68, H; 4.07, N; 4.71; found (%): C; 68.69, H; 4.04, N; 4.71; melting point: 175 °C.

*3-((2-chlorobenzylamino)methylene)-3H-chromene-2,4-dione* (**4g**)

Yield: 79%; Pink amorphous solid; FTIR (KBr, cm^−1^): 3250 (N-H), 1726 (C=O), 1655 (C=C); λ_max_ (cm^−1^): 19,410; ^1^H-NMR(300 MHz, DMSO-d_6_) δ (ppm): 4.80 (d, *J*= 6.0 Hz, 2H, CH_2_ (E)), 4.84 (d, *J* = 6.0 Hz, 2H, CH_2_ (Z)), 7.23–7.36 (m, 2H, Ar-H), 7.38–3.55 (m, 3H, Ar-H), 7.57–7.58 (m, 1H, Ar-H),7.58 (m, 1H, Ar-H), 8.01 (dd *J*_5,6_ = 9.0 Hz, *J*_5,7_ = 3.0 Hz,1H, Ar-H (E)), 8.04 (dd *J*_5,6_ = 9.0 Hz, *J*_5,7_ = 3.0 Hz, 1H, Ar-H (Z)), 8.52 (d, *J*_H(9),NH_ = 12 Hz, C_9_-H (E)), 8.67 (d, *J*_H(9),NH_ = 15 Hz, C_9_-H (Z)), 10.50 (s, br, 1H, NH (Z)), 12.19 (s, br, 1H, NH (E)) (Figure S7A); ^13^C-NMR (300 MHz, CDCl_3_): 52.45, 52.67, 97.49, 117.32, 120.50, 124.01, 124.21, 125.73, 126.43, 127.68, 129.95, 130.24, 130.49, 132.40, 134.44, 154.92, 160.95, 162.52 (Figure S7B); EI-MS, *m*/*z* (%): 313(M^+^ 100), 296(4.1), 278(11.0), 257(8.9), 249(2.3), 188(7.1), 176(18.4), 158(5.6), 140(9.9), 125(38.7), 89(5.6), 63(7.3), 44(29.2); elemental analysis: C_17_H_12_NO_3_Cl: (313.74); calculated (%): C; 65.08, H; 3.86, N; 4.46; found (%): C; 65.07, H; 3.83, N; 4.46; melting point: 165 °C.

*3-((3-chlorobenzylamino)methylene)-3H-chromene-2,4-dione* (**4h**)

Yield: 77%; Light purple crystalline solid; FTIR (KBr, cm^−1^): 3233 (N-H), 1703 (C=O), 1630 (C=C); λ_max_ (cm^−1^): 19,550; ^1^H-NMR: (300 MHz, DMSO-d_6_) δ (ppm): 4.69 (d, *J* = 6.0 Hz, 2H, CH_2_ (E), 4.74 (d, *J* = 9.0 Hz, 2H, CH_2_ (Z), 7.20–7.37 (m, 2H, Ar-H), 7.56–7.59 (m, 4 H, Ar-H), 7.59 (m, 1H, Ar-H), 8.01 (dd *J*_5,6_ = 6.0 Hz, *J*_5,7_ = 3.0 Hz, 1H, Ar-H (E)), 8.03 (dd *J*_5,6_ = 6.0 Hz, *J*_5,7_ = 3.0 Hz, 1H, Ar-H (Z)), 8.51 (d, *J*_H(9),NH_ = 15 Hz, C_9_-H (E)), 8.66 (d, *J*_H(9),NH_ = 15 Hz, C_9_-H (Z)), 10.48 (s, br, 1H, NH (Z)), 12.15 (s, br, 1H, NH (E)) (Figure S8A); ^13^C-NMR (300 MHz, CDCl_3_): 53.80, 53.96, 117.31, 117.36, 120.45, 124.07, 124.28, 125.73, 126.44, 127.84, 129.09, 130.61, 134.55, 136.58, 154.93, 160.83, 162.42 (Figure S8A); EI-MS, *m*/*z* (%): 293(M^+^ 100), 296(15.5), 278(26.2), 249(1.6), 219(2.6), 188(68.9), 176(54.4), 164(7.7), 146(4.6), 139(7.6), 125(48.4),105(1.9), 82.9(28.5), 89(11.1), 63(6.4), 44(14.4); elemental analysis: C_17_H_12_NO_3_Cl: (313.74); calculated (%): C; 65.08, H; 3.86, N; 4.46; found (%): C; 65.07, H; 3.83, N; 4.46; melting point: 176 °C.

*3-((4-chlorobenzylamino)methylene)-3H-chromene-2,4-dione* (**4i**)

Yield: 79%; Light pink crystalline solid; FTIR (KBr, cm^−1^): 3200 (N-H), 1715 (C=O), 1636 (C=C); λ_max_ (cm^−1^): 19,608; ^1^H-NMR(300 MHz, DMSO-d_6_) δ (ppm): 4.71(d, *J* = 6.0 Hz, 2H, CH_2_ (E)), 4.73 (d, *J* = 9.0 Hz, 2H, CH_2_ (Z)), 7.23–7.42 (m, 4 H, ph), 7.56–7.59 (m, 2H, C_6_-H & C_8_-H),7.61 (m, 1H, C_7_-H), 8.00 (dd *J*_5,6_ = 6.0 Hz, *J*_5,7_ = 3.0 Hz, 1H, C_5_-H (E)), 8.02 (dd *J*_5,6_ = 6.0 Hz, *J*_5,7_ = 3.0 Hz, 1H, C_5_-H (Z)), 8.50 (d, *J*_H(9), NH_ = 15 Hz, C_9_-H (E)), 8.65 (d, *J*_H(9), NH_ = 12 Hz, C_9_-H (Z)), 10.47 (s (br.), 1H, NH (Z)), 12.14 (s (br.), 1H, NH (E)) (Figure S9A); ^13^C-NMR (300 MHz, CDCl_3_): 53.75, 53.88, 97.51, 117.35, 120.45, 124.07, 124.28, 125.73, 126.43, 129.05, 129.53, 133.05, 134.53, 160.71, 162.30 (Figure S9B); EI-MS, *m*/*z* (%): 313(M^+^ 100), 296(13.8), 278(2.1), 219(1.7), 188(43.5), 176(32.4), 164(5.0),146(3.4), 138(6.8), 125(81.7), 105(1.8), 89(9.6), 63(4.6),51(2.5), 44(9.7); elemental analysis: C_17_H_12_NO_3_Cl: (313.74); calculated (%): C; 65.08, H; 3.86, N; 4.46; found (%): C; 65.07, H; 3.83, N; 4.46; melting point: 166 °C.

### 3.3. Proposed Mechanism of Reaction

**Figure 6:**
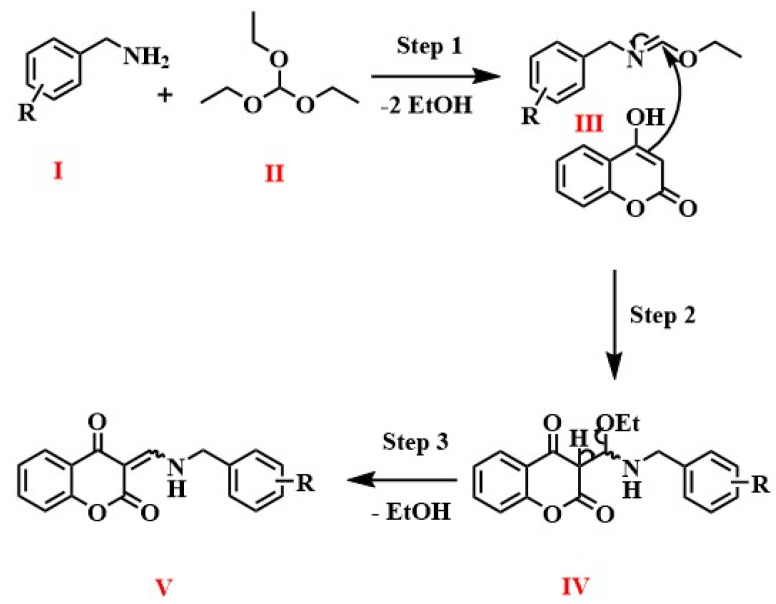
Proposed mechanism for the synthesis of enamines from 4-hyroxycoumarin. Benzylamine (**I**), triethyl orthoformate (**II**) the imine intermediate (**III**), aminal (**IV**), enamine (**V**).

For insight into the chemical reaction, a mechanism was proposed to form the targeted product, as shown in Figure 6 [27]. Briefly, in the first step, the nucleophilic addition of benzylamine (**I**) to triethyl orthoformate (**II**) occurs, releasing two molecules of ethanol. In the second step, the imine intermediate (**III**) reacts with a molecule of 4-hydroxycoumarin to produce aminal (**IV**). In the third and final step, another ethanol molecule is eliminated from aminal (**IV**), forming the final product, enamine (**V**).

### 3.4. Potato Disc Tumor Assay

*Agrobacterium tumefaciens* was kept in nutrient agar for 48 h before use in the assay. *A. tumefaciens* were standardized to 1 × 10^9^ CFU in phosphate-buffered saline. Fresh potatoes purchased from the local market of Sargodha, Pakistan, were washed several times with distilled water and bleach to remove impurities on the surface. The potato skin was peeled off after cleaning the surface with a sterile towel. After peeling, potatoes were washed with distilled water and cut into small discs of 0.5 cm in diameter. These discs were placed in a 24-well culture plate containing 15% agar in water. Dilute solutions of the as-synthesized compounds containing both the isomers (‘E’ and ‘Z’ isomer) (**4a**–**4i**) in the range of 0.1–50 mg/mL were prepared in DMSO and 50 μL of each solution was applied on potato disc. Pure DMSO was used as a negative control, and vinblastine (standard drug) solutions in DMSO were used as a positive control. Vinblastine was used as a positive control due to its almost similar mode of action as the synthesized compounds (**4a**–**4i**); it binds to the microtubular proteins to hinder the cell division of cancer cells [35]. After incubation for 14 days, discs were stained with Lugol’s reagent. This reagent stained the starch in the discs to blue, but the tumors appeared creamy. Tumors were counted under a microscope in each disc to calculate the IC_50_ values.

The bacterial viability of compounds was also checked by the disc diffusion method by loading sterile paper discs with compounds (0.1 mg/mL). Pure DMSO was used as a positive control. After incubation of 48 h, the growth of bacteria was evident, which showed the non-active nature of the compounds at this concentration (0.1 mg/mL) toward bacteria.

### 3.5. Molecular Docking Analysis

**Figure 7:**
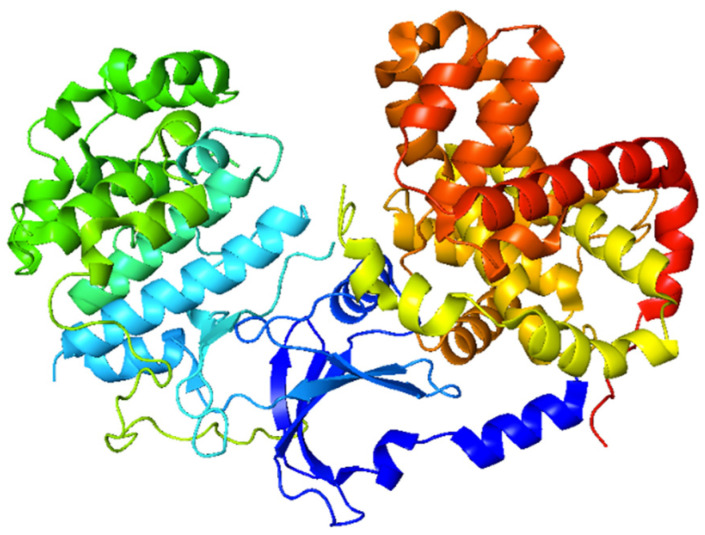
Crystal structure of CDK-8 enzyme.

The in silico anti-tumor activity of synthesized compounds was predicted by screening against the CDK-8 protein using a YASARA (Yet Another Scientific Artificial Real Application) software version 20.7.4 [40]. The 3D structure of CDK-8, as shown in Figure 7, was obtained from Protein Data Bank (PDB ID, 5FGK). AutoDock LGA module and AMBER03 forcefields in YASARA with 100 global docking runs and 1000 random seed values were used for docking. The AutoDock local search (LGA-LS) method in YASARA was then used to rescore docked ligands (**4a**–**4i**), as described before [41].

The docking scoring and rescoring were calculated following the empirical Equation (1).

(1) $$\Delta G={\Delta G}_{\left(Van\;der\;Waal\right)}+{\Delta G}_{\left(H-bonding\right)}+{\Delta G}_{\left(electrostatic\right)}+{\Delta G}_{\left(torsional\;free\;energy\right)}+{\Delta G}_{\left(desolvation\;energy\right)}$$

The LigPlus [42] and PyMOL [43] were used to extract the lowest energy binding energy docking poses, and ligand-protein interactions were mapped.

### 3.6. Statistical Analysis

Each bioactivity experiment was repeated thrice, and results were analyzed statistically by ANOVA. Statistical significance was accepted at a level of *p* < 0.05. Mean ± SD values were reported in tables and graphs.

## 4. Conclusions

Novel 3-formyl-4-hydroxycoumarin-derived enamines (**4a**–**4i**) were synthesized by a one-pot condensation reaction and characterized by state-of-the-art spectroscopic and analytical techniques. All the compounds were evaluated for their anti-tumor potential against *Agrobacterium tumefaciens* by potato disc tumor assay. The bacterial viability of compounds was also checked, which showed no prominent effect on bacterial growth. Five compounds (**4a**, **4c**, **4e**, **4g**, and **4h**) have shown better activity than the standard drug, vinblastine. Molecular docking simulations of compounds were also performed with the human protein CDK-8. This enzyme is actively involved in the cell cycle, and the synthesized compounds have shown good binding affinities with this protein. Molecular docking results were in good agreement with the experimental IC_50_ values of the compounds.

## Figures and Tables

**Table 1:** Optimization of reaction conditions of enamine synthesis.

Sr. No.	Solvents	Catalysts	Reaction Conditions	Yield %
1	MeOH	-	Reflux, 3 h	80
2	EtOH	-	Reflux, 3 h	75
3	THF	-	Reflux, 3 h	65
4	CHCl_3_	-	Reflux, 3 h	64
5	2-BuOH	-	Reflux, 3 h	94
6	2-BuOH	-	Reflux, 6 h	78
7	2-BuOH	AcOH	Reflux, 3 h	55
8	2-BuOH	ZnCl_2_	Reflux, 3 h	72
9	2-BuOH	AlCl_3_	Reflux, 3 h	74
10	2-BuOH	(CH_3_COO)_2_Pb	Reflux, 3 h	71
11	2-BuOH	Net_3_	Reflux, 3 h	60
12	2-BuOH	HCl	Reflux, 3 h	52

**Table 2:** Binding energies, dissociation constants of synthesized compounds, native ligands, and vinblastine.

Sr. No.	Compound	Protein	Binding Energy (kcal/mol)	Dissociation Constant (nM)	Main Contacting Amino Acid Residues
1	** 4a **	CDK-8	−9.22	175.54	Lys-47, Asp-98, Gly-161 & Glu- 101
2	** 4b **		−8.45	89.46	Lys-47, Tyr-99, Gly-161 & Glu-101
3	** 4c **		−9.41	444.39	His-154, Pro-194 & Phe-195,
4	** 4d **		−8.87	194.89	Lys-47, Asp-48, Tyr-99, & Gly-161
5	** 4e **		−9.33	192.9	Lys-47, Tyr-99, Gly-161 & Glu-165
6	** 4f **		−8.66	83.5	Tyr-153, Tyr-156, Arg-157 & Pro-158
7	** 4g **		−9.53	106.69	Lys-47, Asp-98, Tyr-99 & Glu- 101
8	** 4h **		−9.52	104.28	Trp-6, Glu-98, His-154 & Pro-194
9	** 4i **		−8.53	98.42	Phe-5, Glu-98, His-154 & Phe-195
10	** Vinblastine **		−9.11	3170	Asp-46, Lys-47, Gly-161 & Lys-355
11	** 5XG **		−9.18	185.8	Val-27, Ala-100, Asp-173 & Arg-356

## Data Availability

The raw spectra and data are available on request.

## Supplementary Materials

The following supporting information can be downloaded at: https://www.mdpi.com/article/10.3390/molecules28155828/s1, Potato disc assay results, ^1^H-NMR spectra, ^13^C-NMR spectra and molecular docking diagrams are given in supporting information.

## Sample Availability

Sample of the compounds are available from authors on request.
