# Supplementary material for: In Silico and In Vitro Studies of 4-Hydroxycoumarin-Based Heterocyclic Enamines as Potential Anti-Tumor Agents
Source: Molecules. 2023 Aug 2;28(15):5828. doi: 10.3390/molecules28155828 (PMC10421012; doi:10.3390/molecules28155828)
Supplement: Supplementary file 1 [file molecules-28-05828-s001.zip › molecules-2488038-supplementary.pdf]

Supplementary material

# In Silico and In Vitro Studies of 4-Hydroxycoumarin-Based Heterocyclic Enamines as Potential Anti-Tumor Agents

**Mediha Assad [1,2], Rizwan Nasir Paracha [3], Abu Bakar Siddique [4], Muhammad Ashraf Shaheen [4,*], Nadeem Ahmad [5], Muhammad Mustaqeem [4], Fariha Kanwal [6], Muhammad Zia Ul Mustafa [4], Muhammad Fayyaz ur Rehman [4], Sumaya Fatima [7] and Changrui Lu ***

1 College of Biological Sciences and Medical Engineering, Donghua University, 2999 North Ren Min Road, Shanghai 201620, China

2 Department of Chemistry, Government Graduate Islamia College for Women Cantt Lahore, Lahore 54000, Pakistan

3 Department of Chemistry, Thal University Bhakkar, Bhakkar 30000, Pakistan

4 Institute of Chemistry, University of Sargodha, Sargodha 40100, Pakistan; abubakar.siddique@uos.edu.pk (A.B.S.)

5 Department of Pharmacy, Comsats University Islamabad, Lahore Campus, Lahore 54000, Pakistan

6 School of Biomedical Engineering, Shanghai Jiaotong University, Shanghai 200030, China

7 Research Center, The Fourth Hospital of Hebei Medical University, Shijiazhuang 050011, China

* Correspondence: ashraf.shaheen@uos.edu.pk (M.A.S.); crlu@dhu.edu.cn (C.L.)

**Table S1.** $IC_{50}$ values of synthesized compounds (3a-i) by potato disc tumor assay.

| Sample | $IC_{50}$ (mg/mL) value | Sample | $IC_{50}$ (mg/mL) value |
|---|---|---|---|
| **3a** | 5±0.5 | **3f** | 27.7±3 |
| **3b** | 37.1±4 | **3g** | 1.12±0.2 |
| **3c** | 2.12±0.3 | **3h** | 1.70±0.3 |
| **3d** | 22.7±3 | **3i** | 29.6±4 |
| **3e** | 2.33±0.2 | **Vinblastine (Standard)** | 7.5±0.6 |

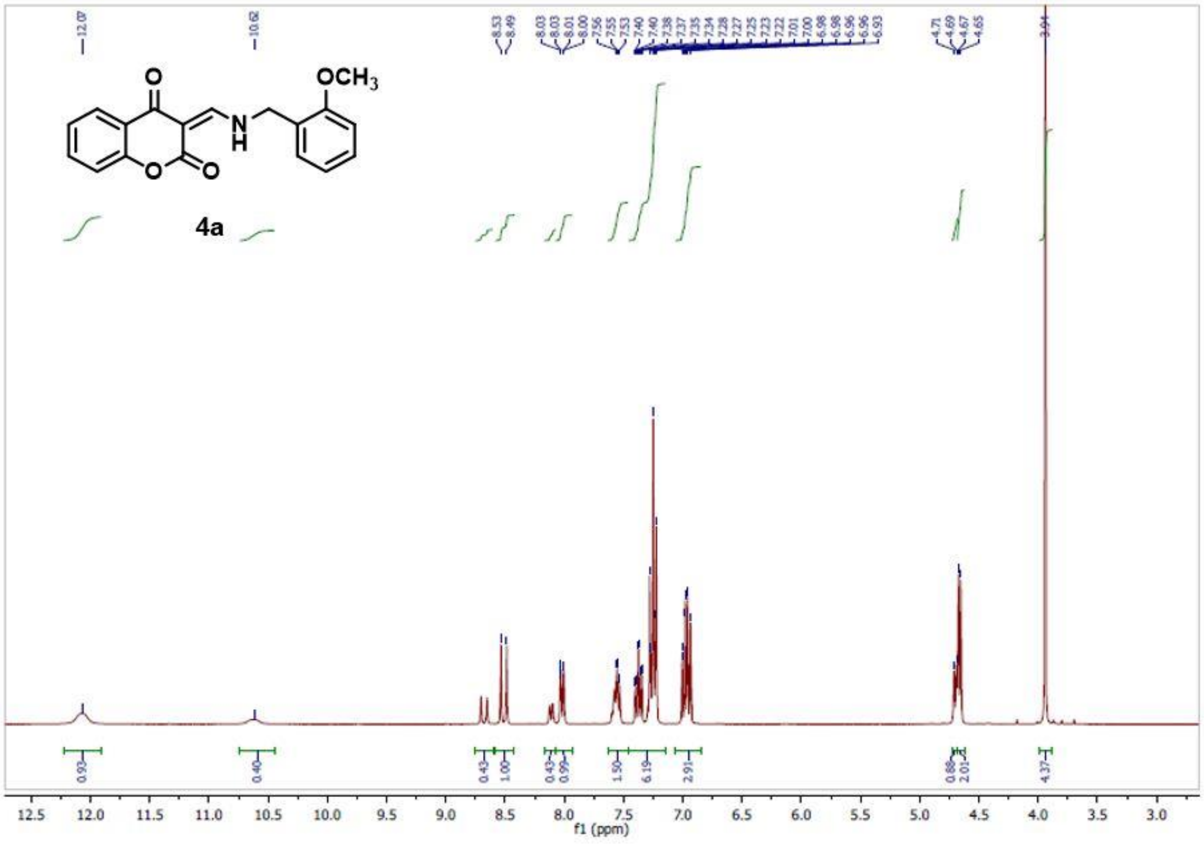


**(A)**

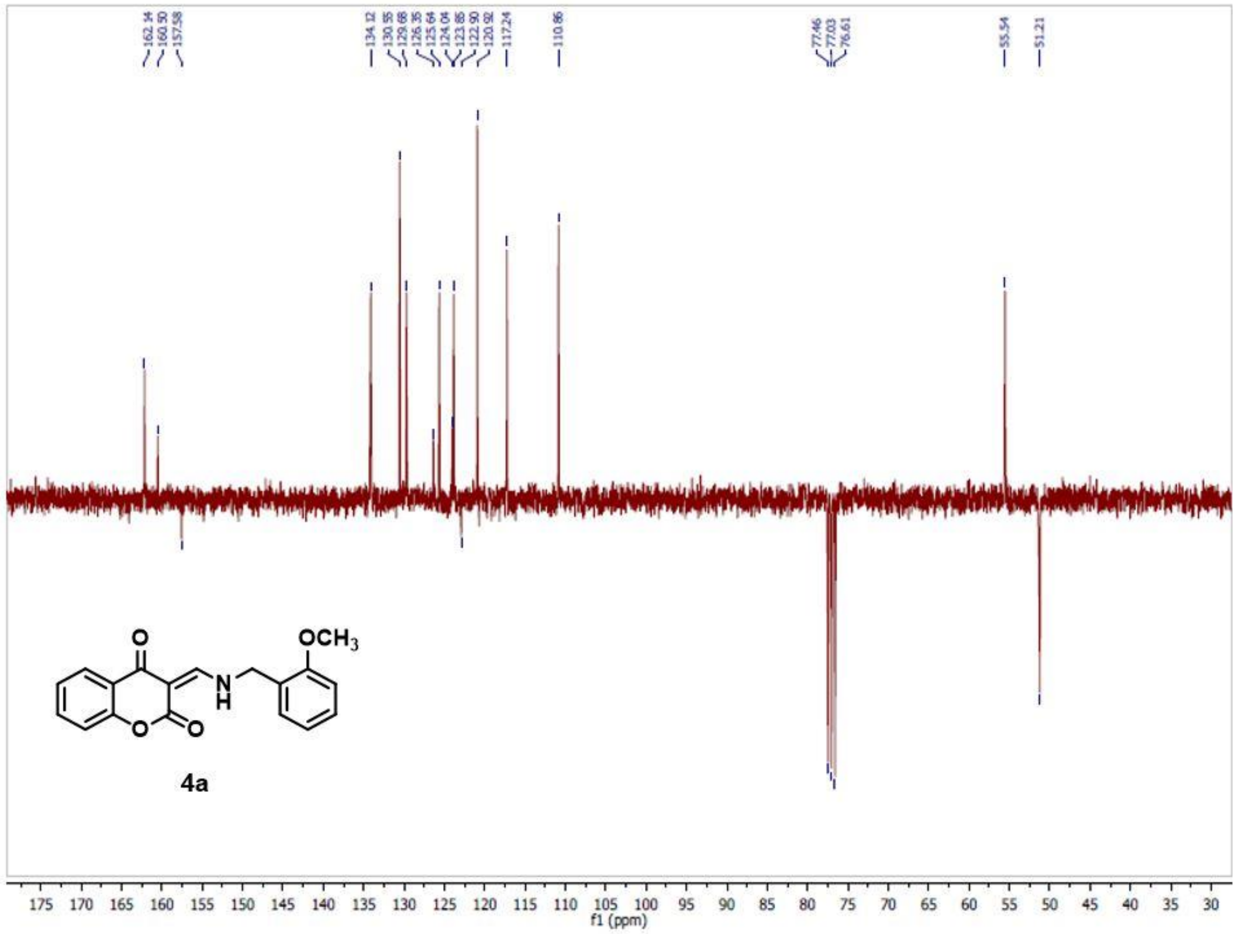

**(B)**

**Figure S1.** (**A**) $^{1}$H-NMR spectrum of **4a.** (**B**) $^{13}$C-NMR spectrum of **4a**.

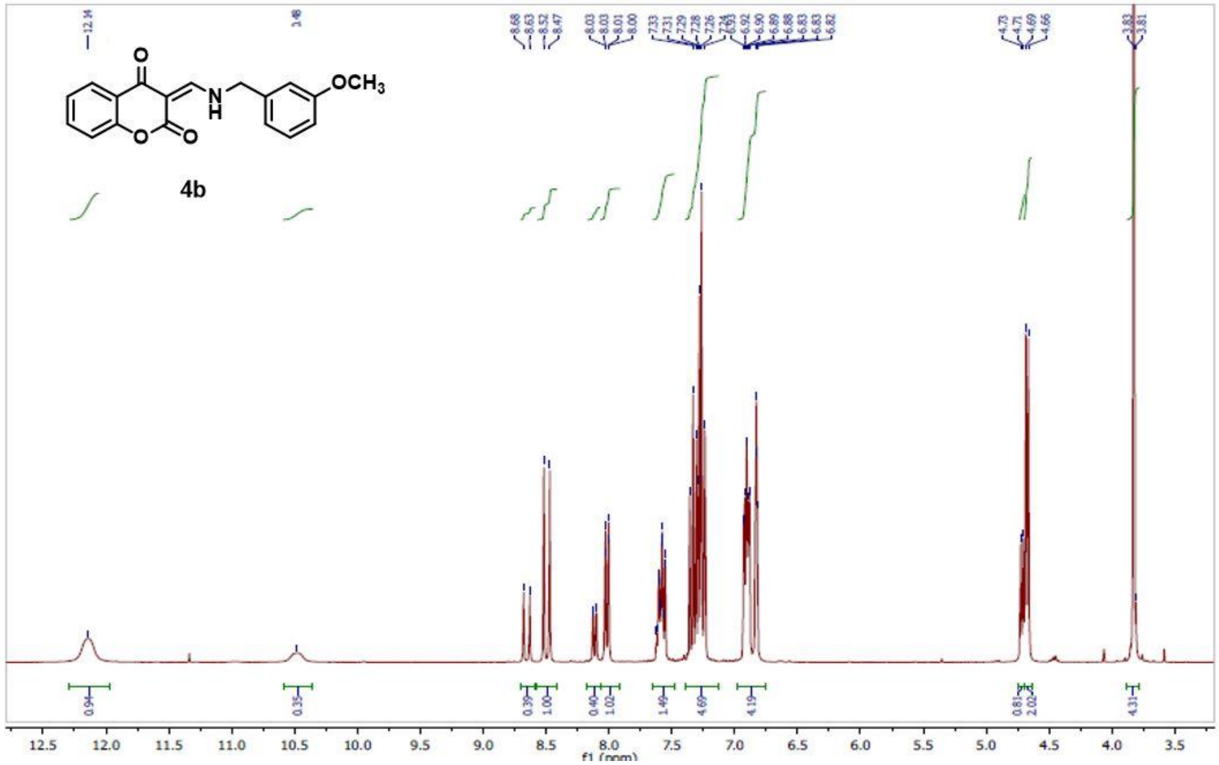

**(A)**

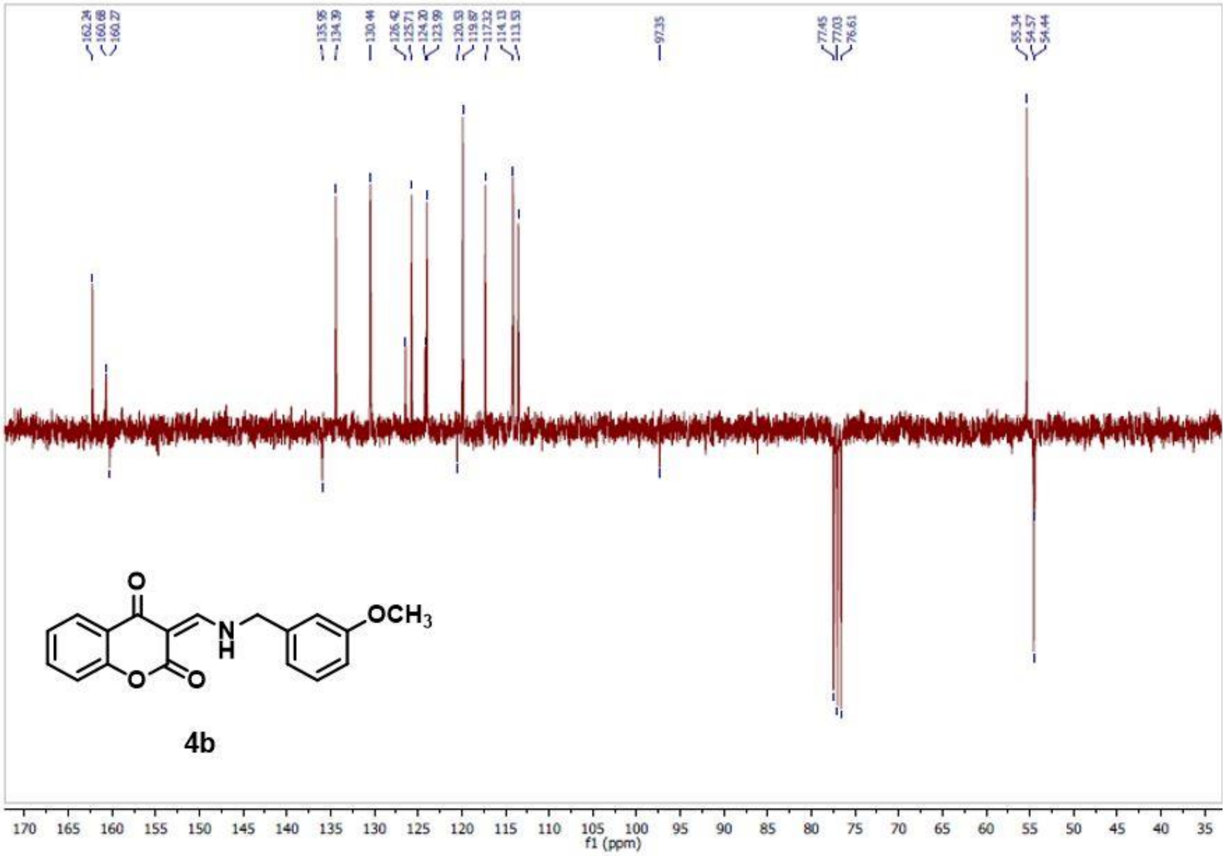

**(B)**

**Figure S2.** (**A**) $^{1}$H-NMR spectrum of **4b.** (**B**) $^{13}$C-NMR spectrum of **4b**.

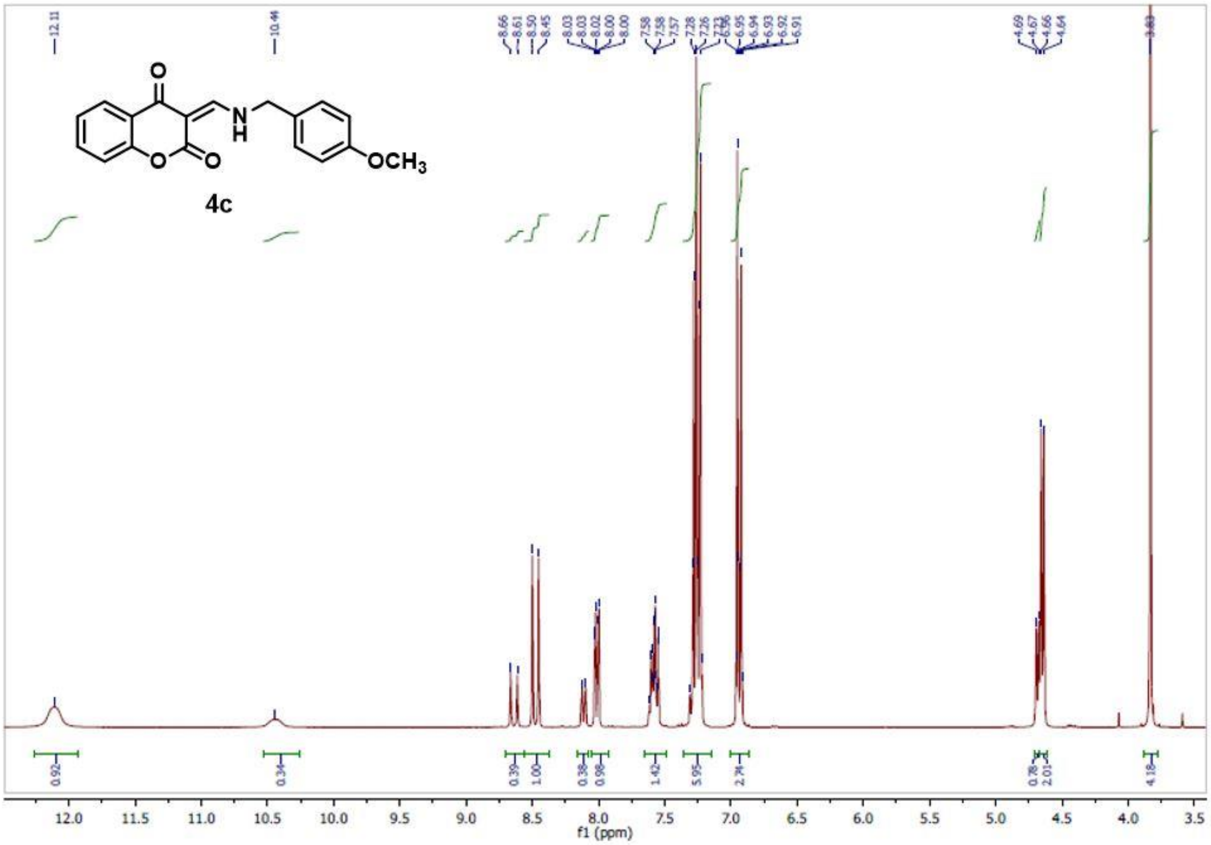


**(A)**

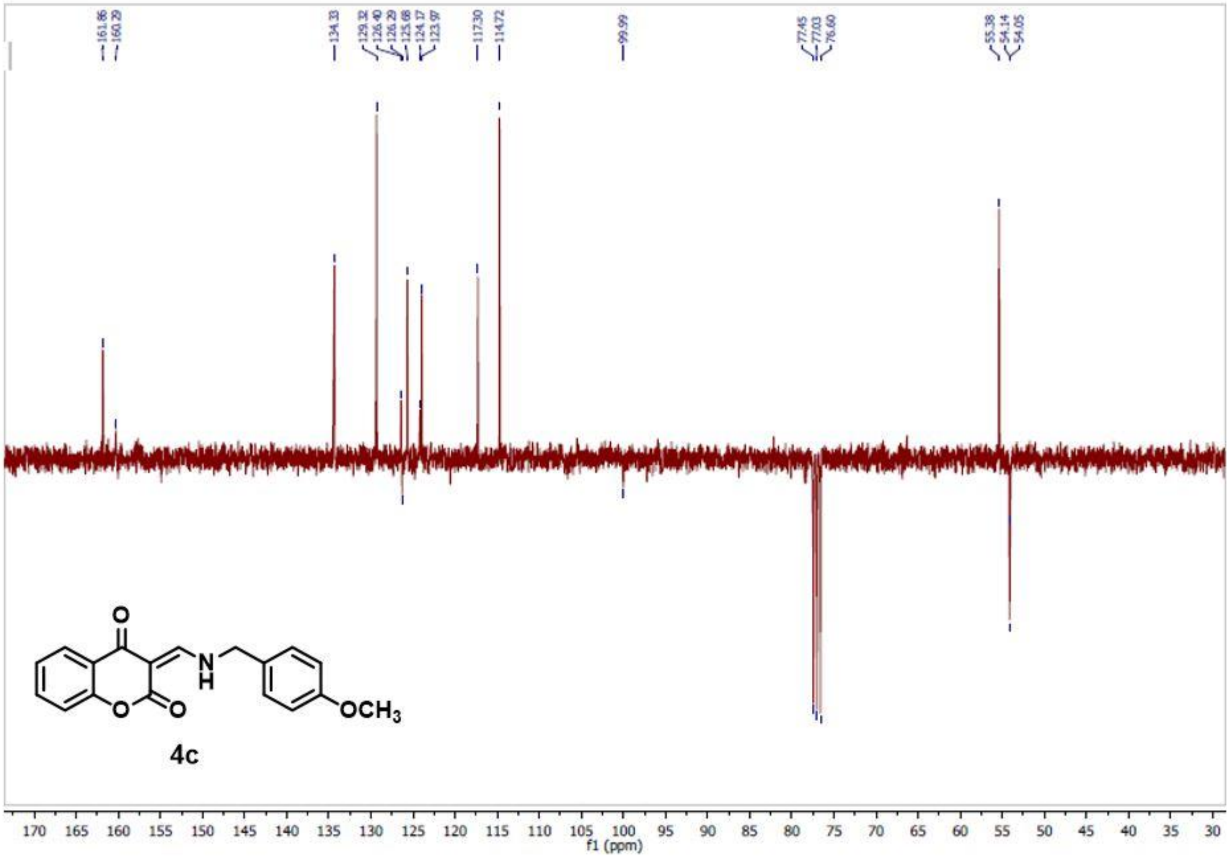

**(B)**

**Figure S3.** (**A**) $^{1}$H-NMR spectrum of **4c**. (**B**) $^{13}$C-NMR spectrum of **4c**.

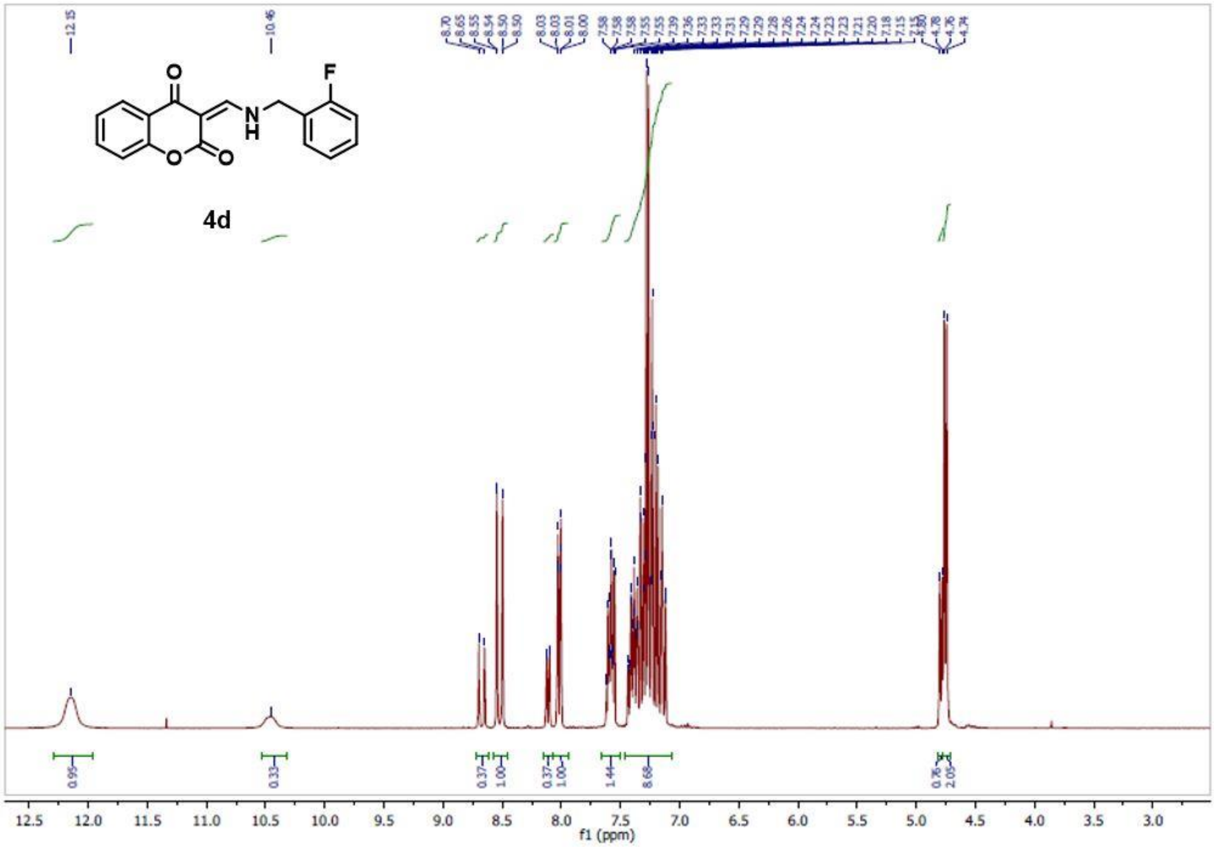

**(A)**

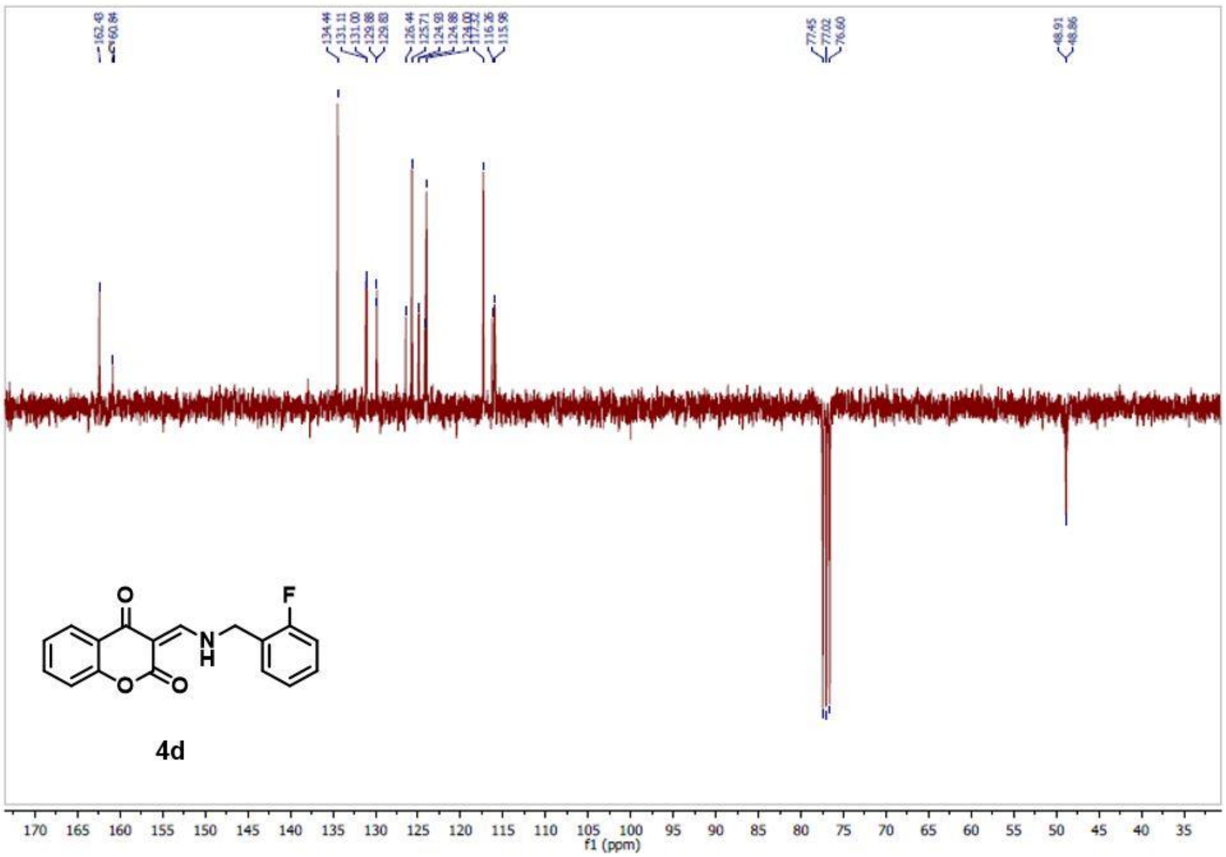

**(B)**

**Figure S4.** (**A**) $^{1}$H-NMR spectrum of **4d.** (**B**) $^{13}$C-NMR spectrum of **4d**.

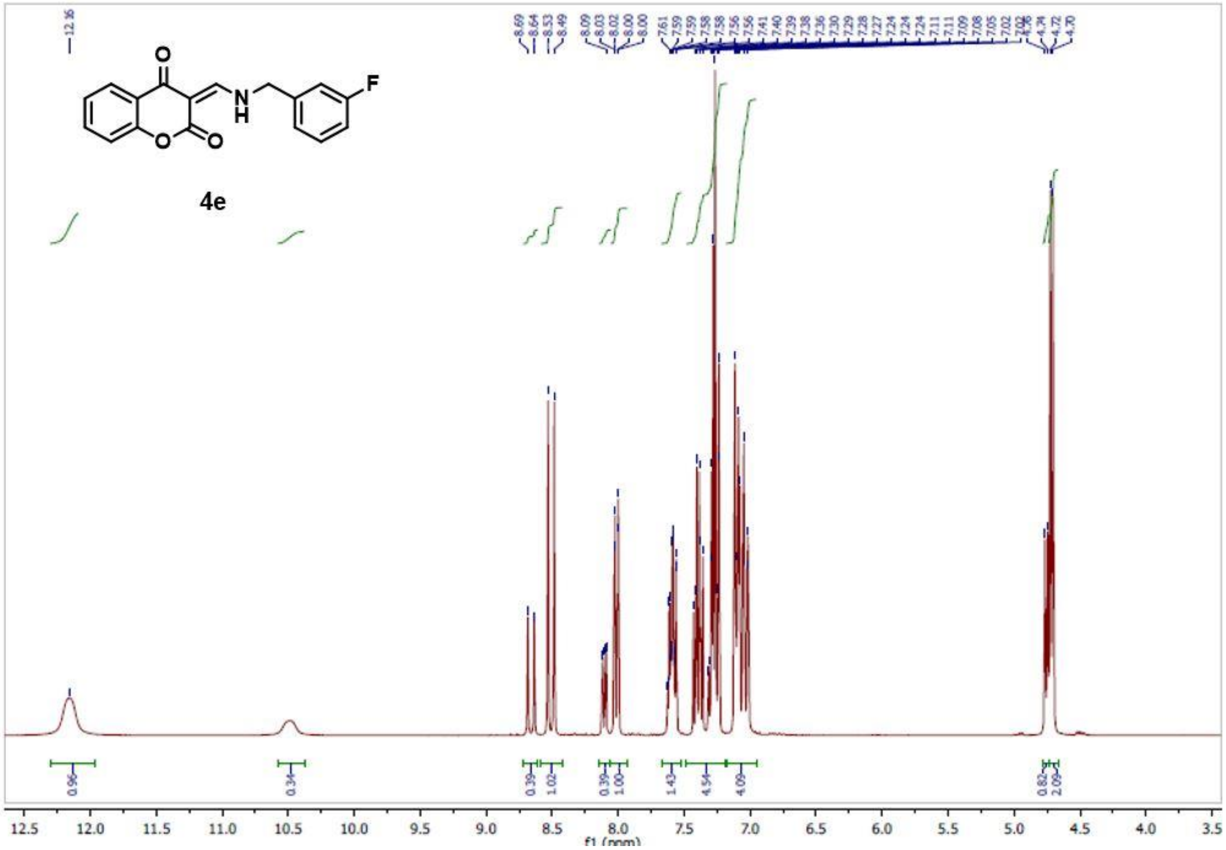

**(A)**

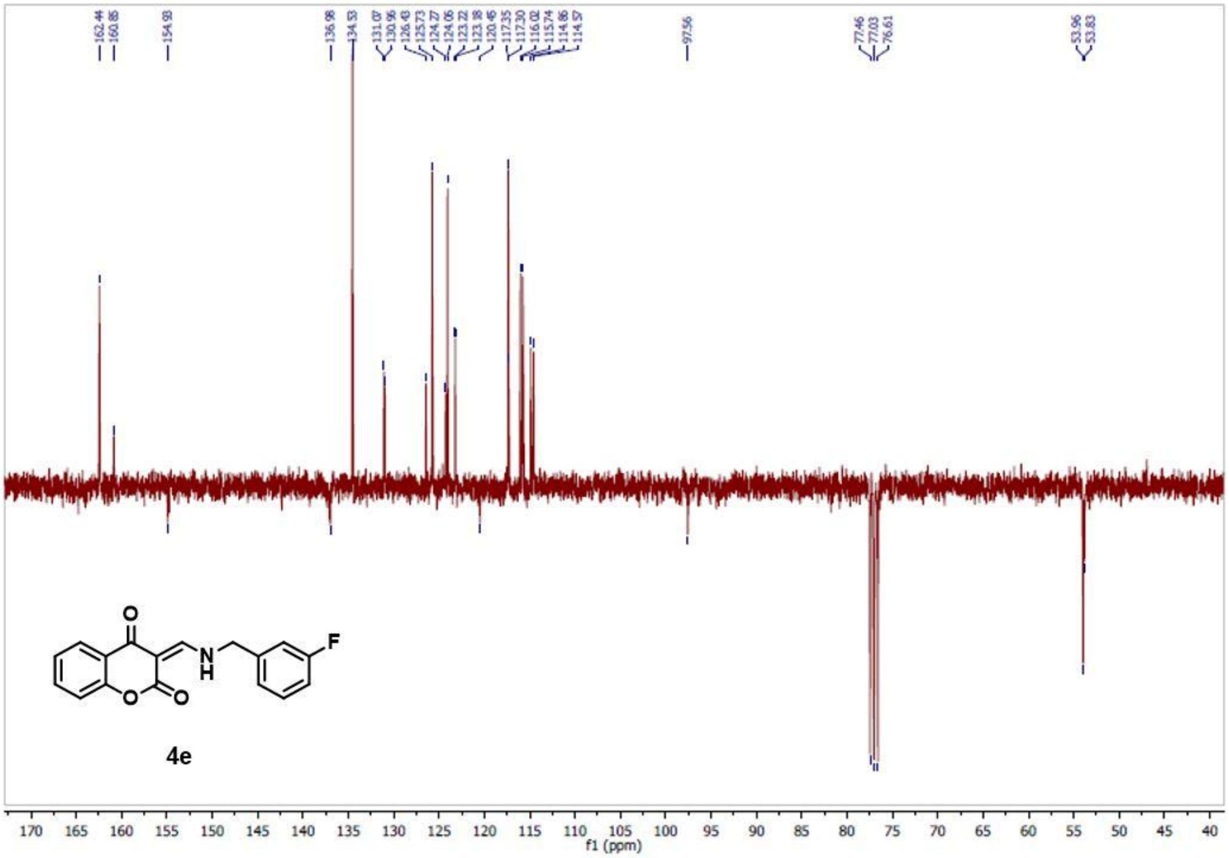


**(B)**

**Figure S5.** (**A**) $^{1}$H-NMR spectrum of **4e.** (**B**) $^{13}$C-NMR spectrum of **4e**.

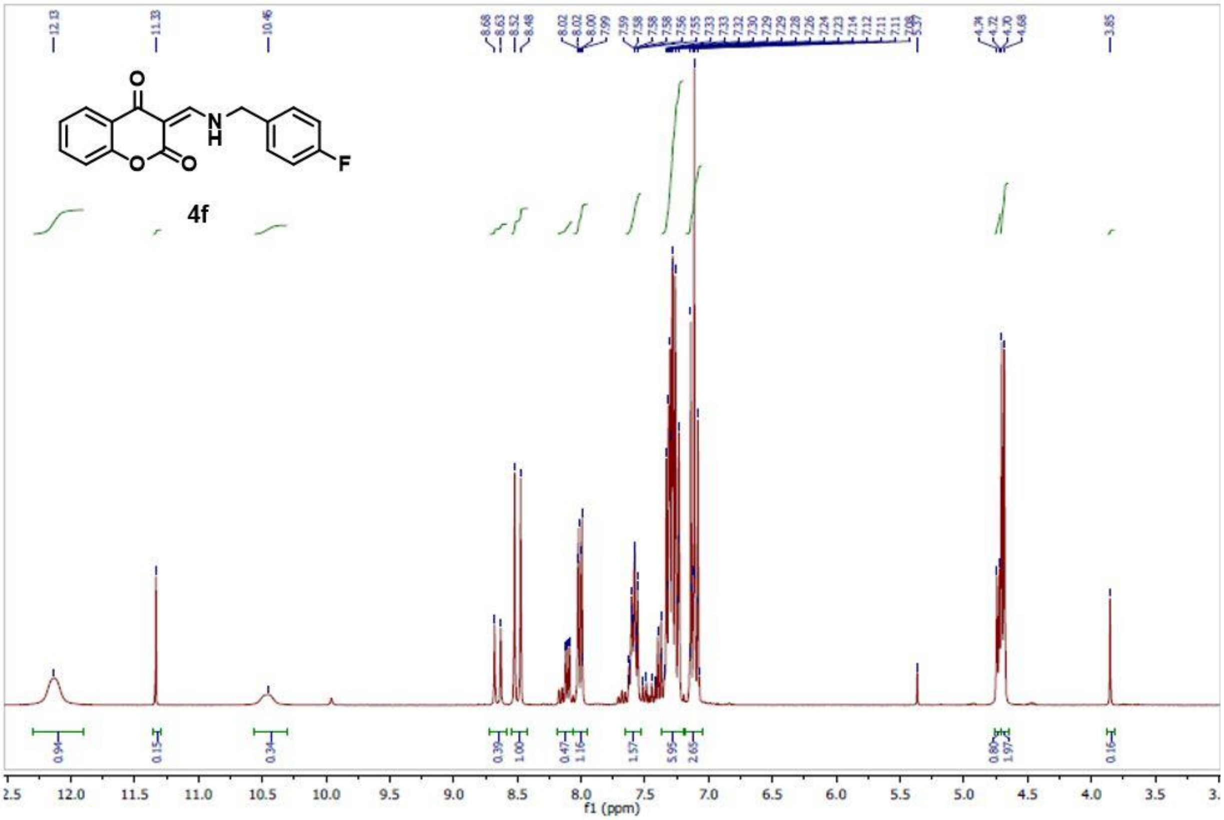


**(A)**

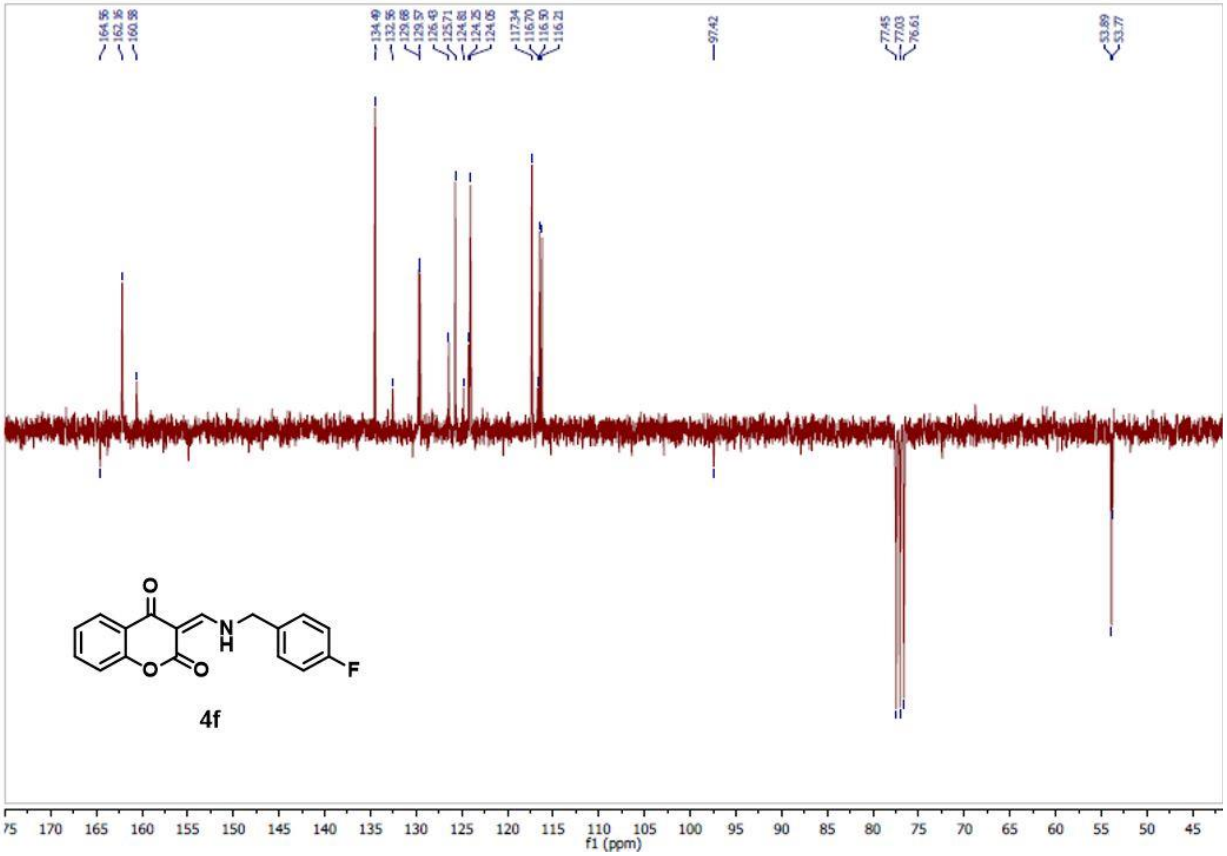

**(B)**

**Figure S6.** (**A**) $^{1}$H-NMR spectrum of **4f.** (**B**) $^{13}$C-NMR spectrum of **4f**.

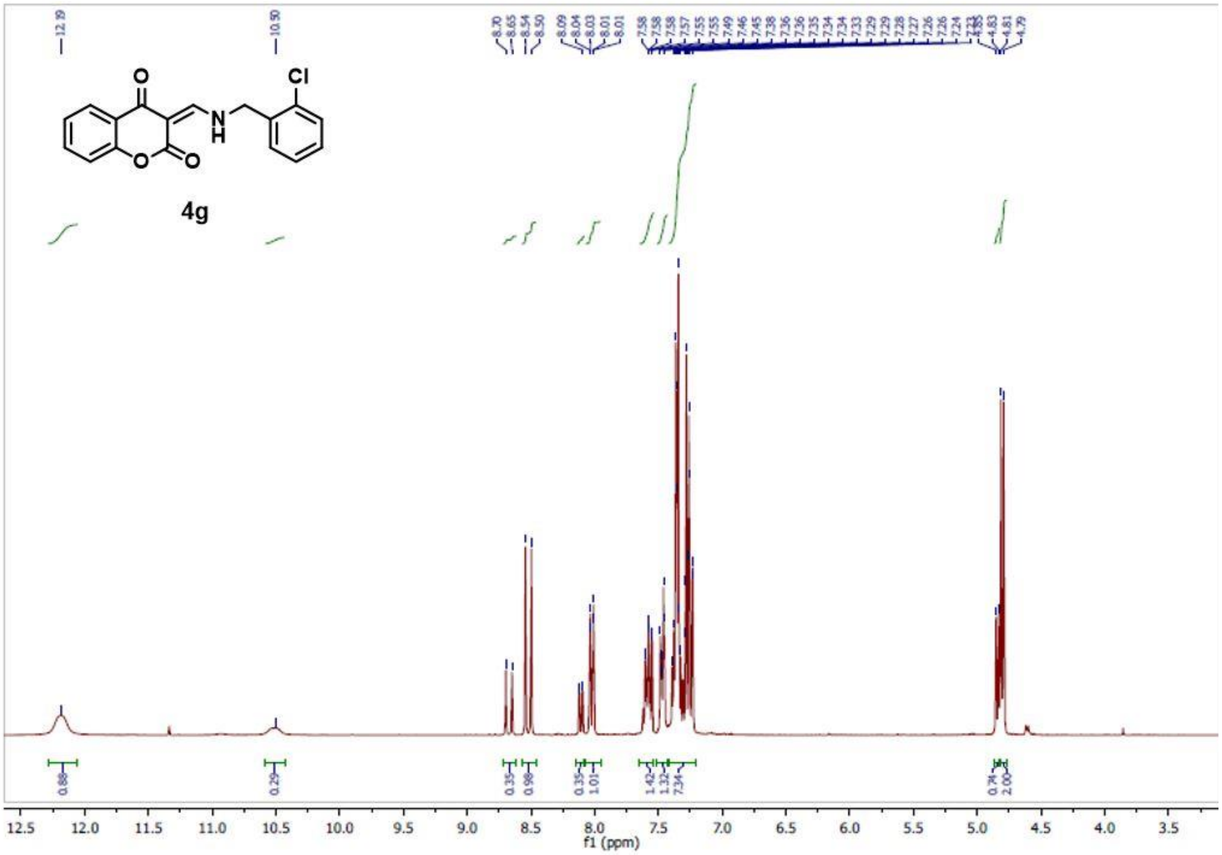

**(A)**

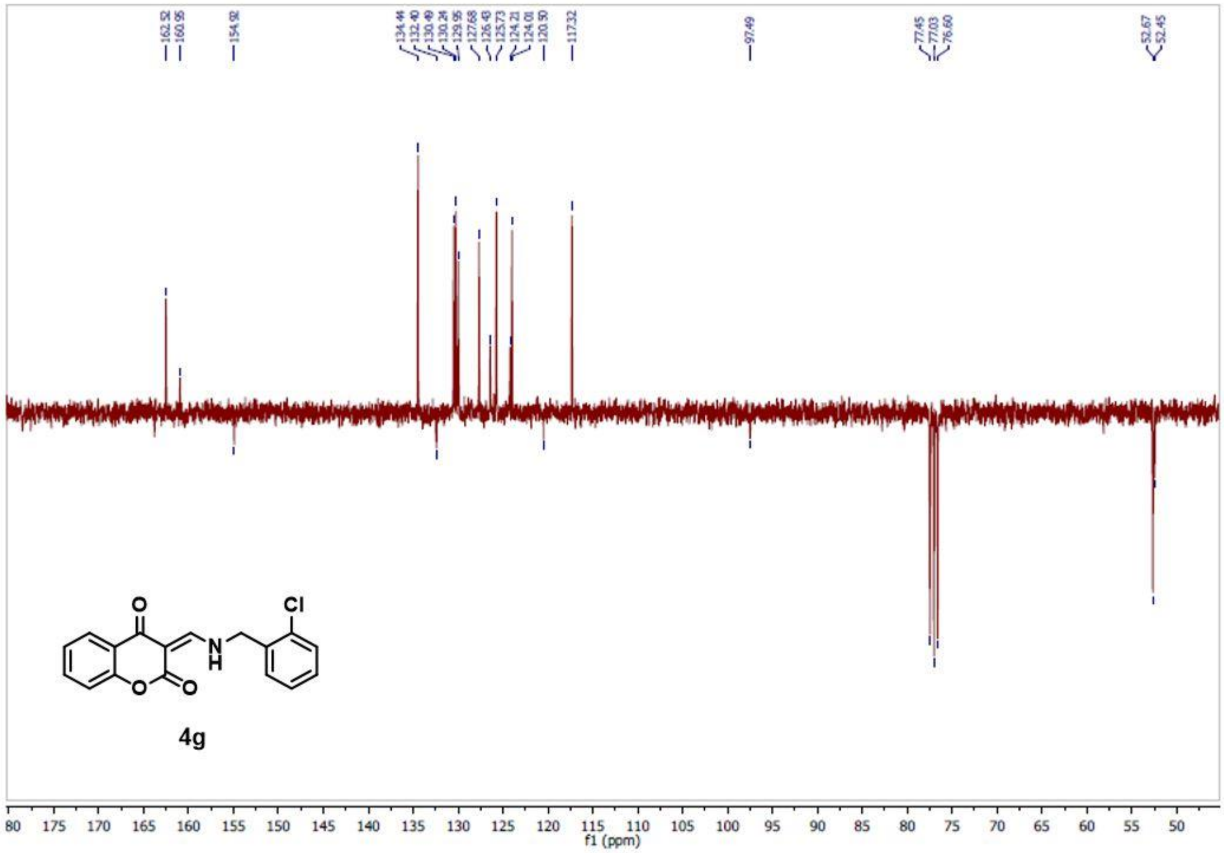


**(B)**

**Figure S7.** (**A**) $^{1}$H-NMR spectrum of **4g**. (**B**) $^{13}$C-NMR spectrum of **4g**.

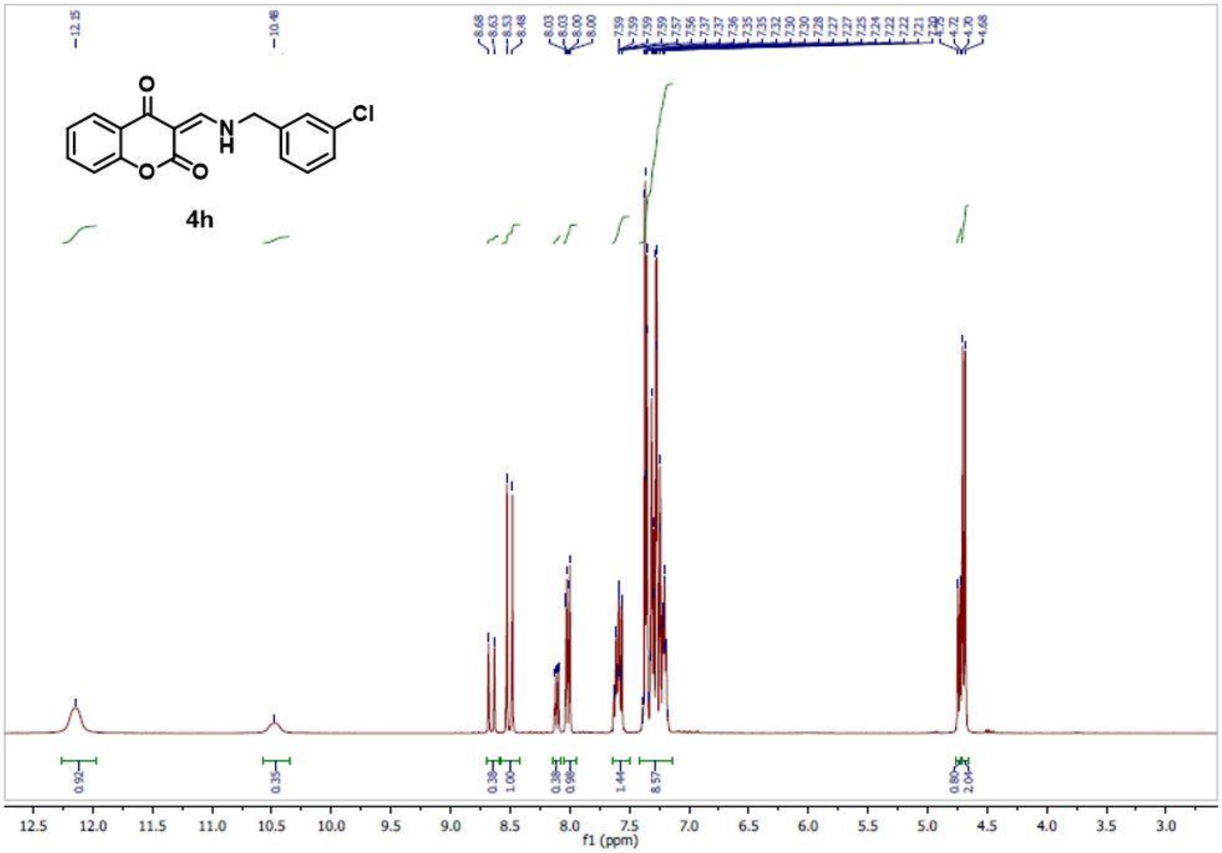


**(A)**

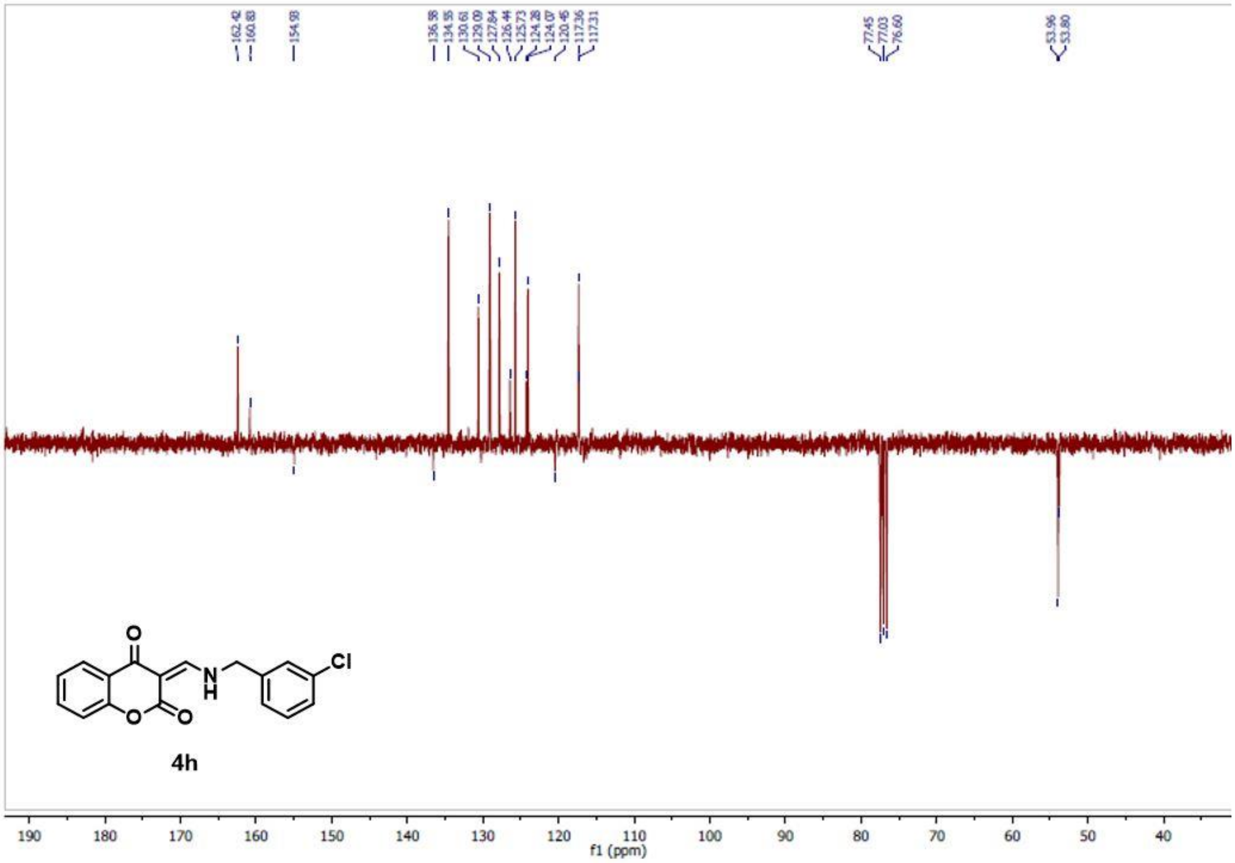


**(B)**

**Figure S8.** (**A**) $^{1}$H-NMR spectrum of **4h**. (**B**) $^{13}$C-NMR spectrum of **4h**.

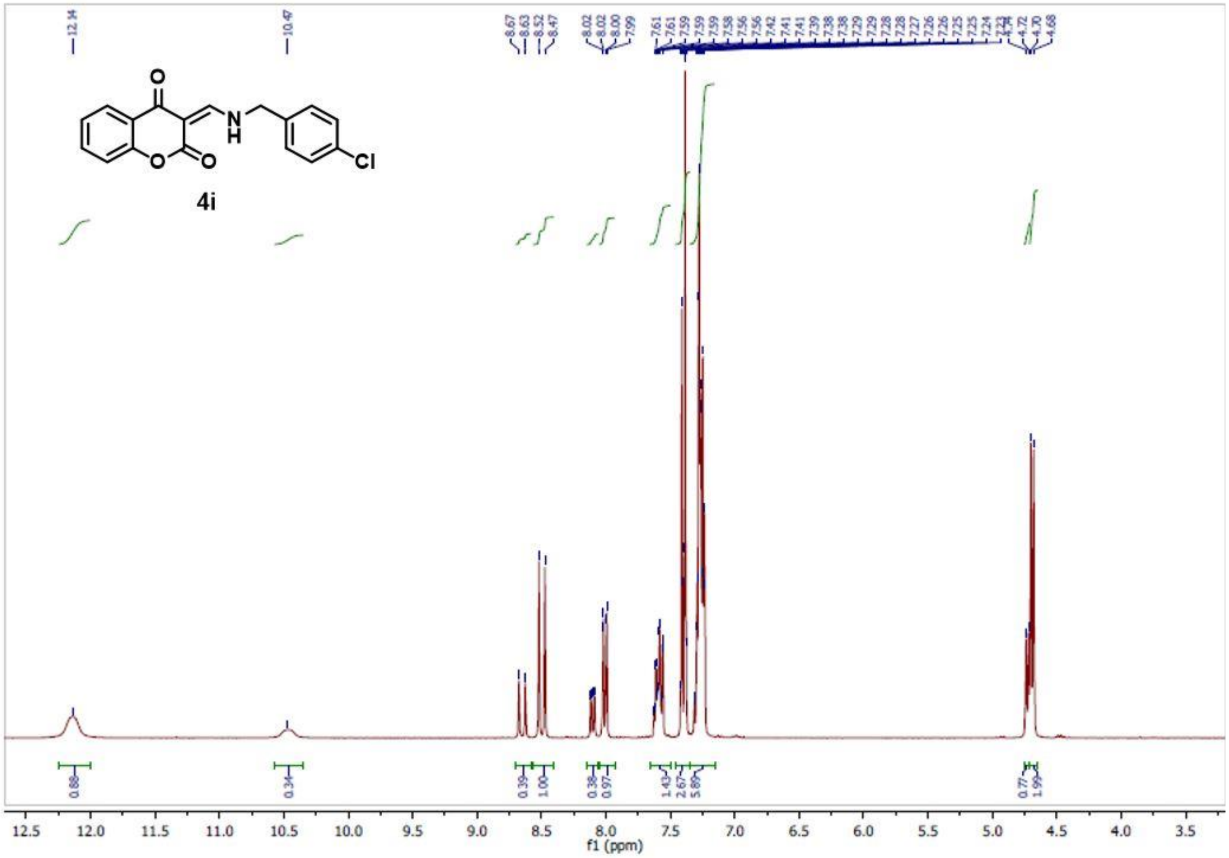

**(A)**

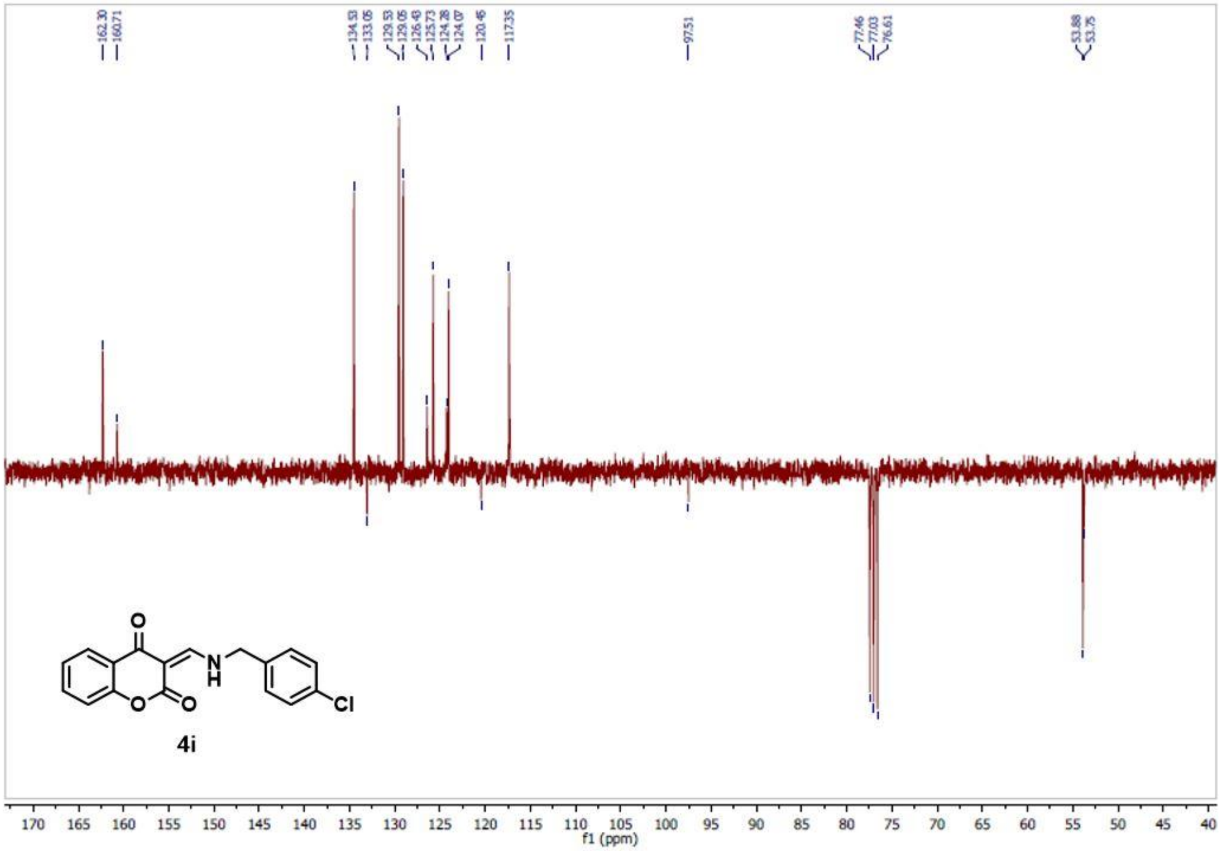


**(B)**

**Figure S9.** (**A**) $^{1}$H-NMR spectrum of **4i**. (**B**) $^{13}$C-NMR spectrum of **4i**.

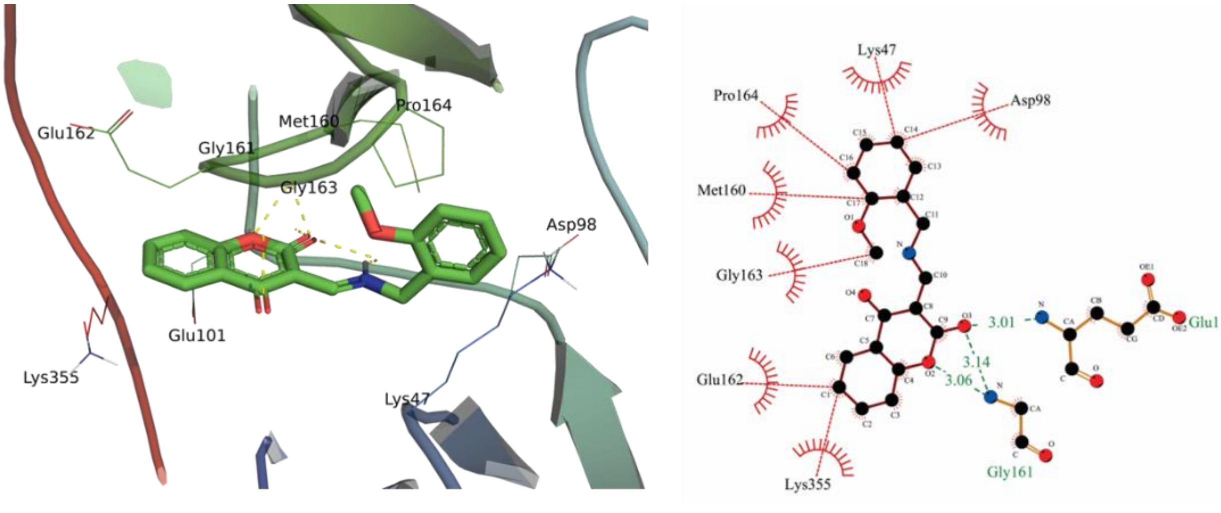


(4**a**)

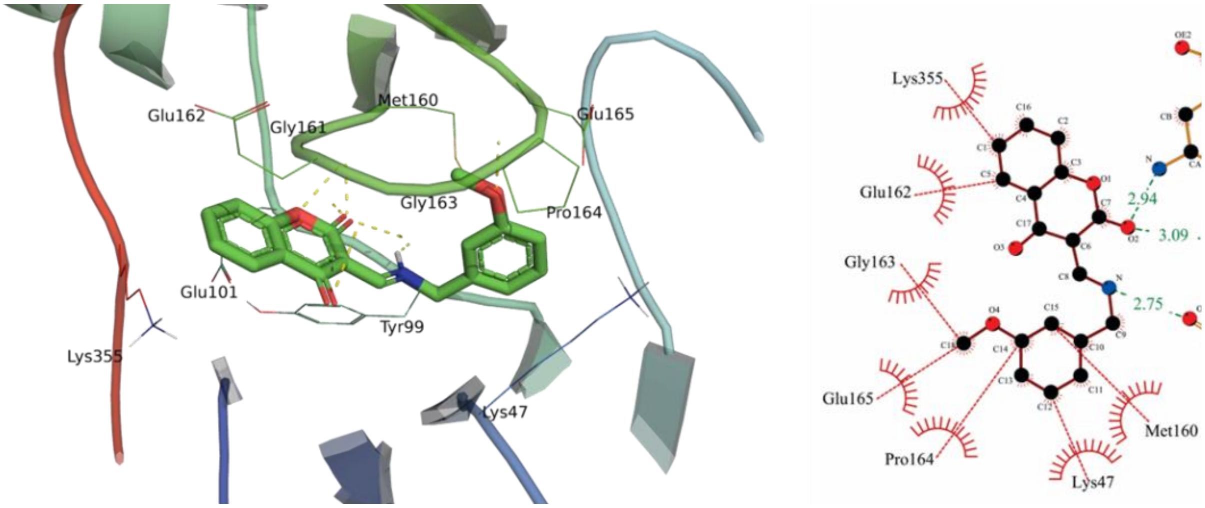
Met160
Glu162
Gly161
Glu165
Gly163
Pro164
Glu101
Tyr99
Lys355
Lys47
Lys355
Glu162
2.94
3.09
Gly163
2.75
Glu165
Met160
Pro164
Lys47

(4**b**)

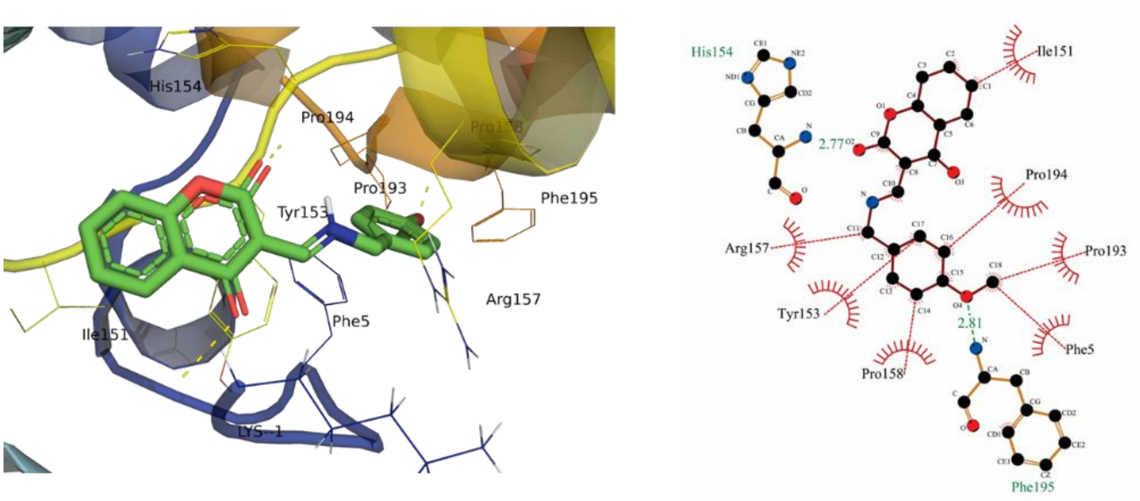
His154
Pro194
Pro193
Phe195
Tyr153
Arg157
Ile151
Phe5
His154
Ile151
2.77
Pro194
Arg157
Pro193
Tyr153
2.81
Phe5
Pro158
Phe195

(4**c**)

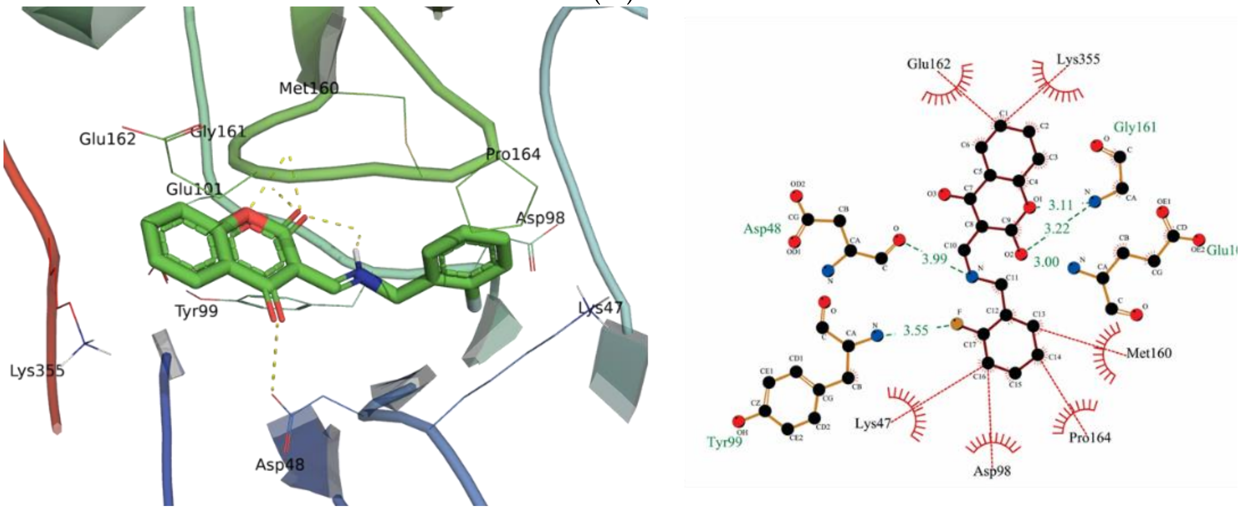
Met160
Glu162
Gly161
Pro164
Glu101
Asp98
Tyr99
Lys47
Lys355
Asp48
Glu162
Lys355
Gly161
Asp48
3.11
3.22
3.99
3.00
3.55
Met160
Lys47
Pro164
Tyr99
Asp98

(4**d**)

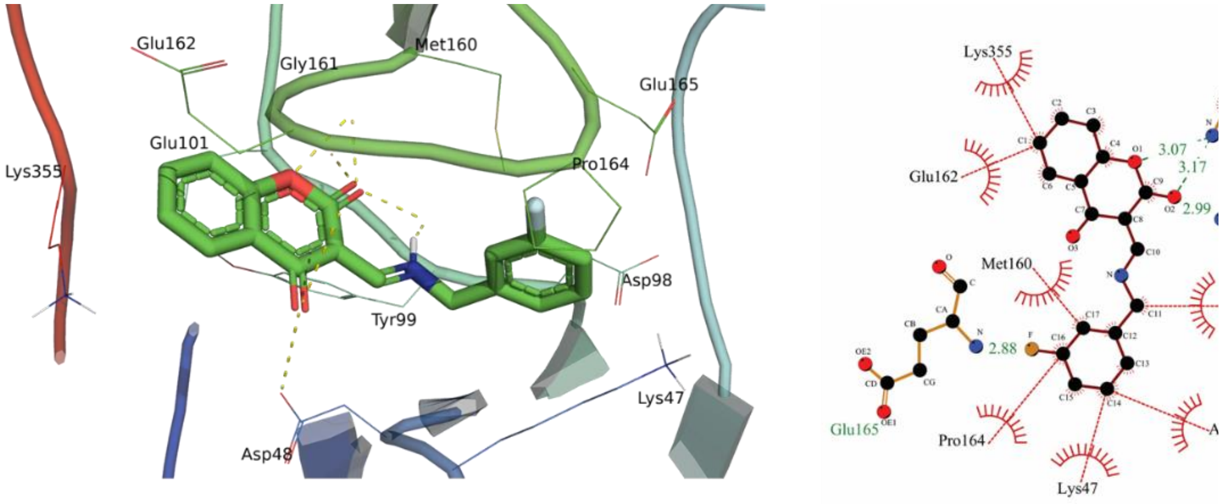

Glu162
Gly161
Met160
Glu165
Glu101
Lys355
Pro164
Asp98
Tyr99
Lys47
Asp48
Lys355
Glu162
3.07
3.17
2.99
Met160
2.88
Glu165
Pro164
Lys47


(4e)

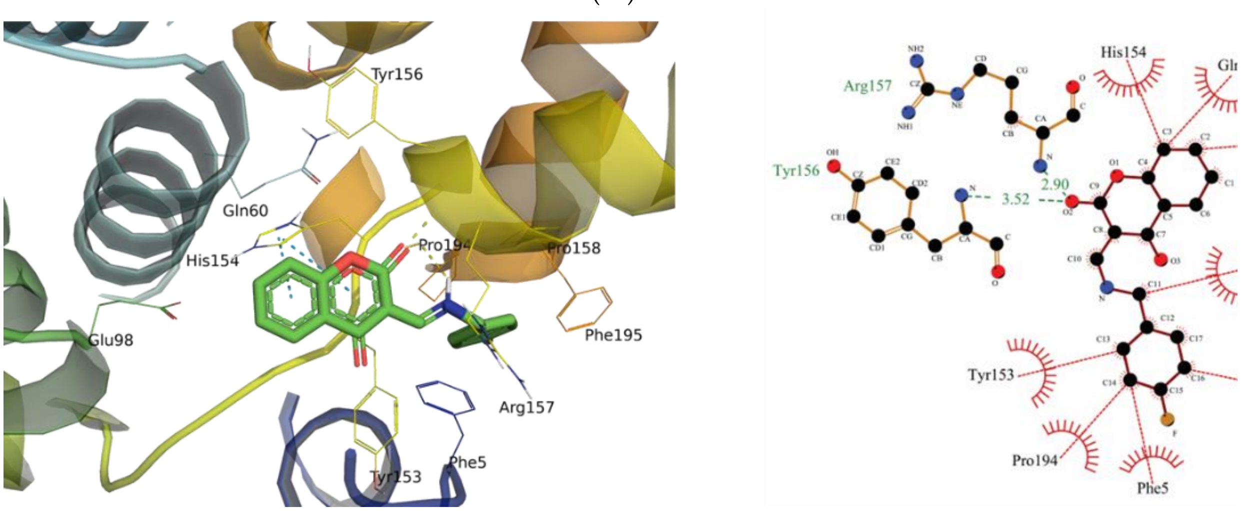

Tyr156
Gln60
His154
Pro158
Glu98
Phe195
Arg157
Phe5
Tyr153
His154
Arg157
Tyr156
2.90
3.52
Tyr153
Pro194
Phe5


(4f)

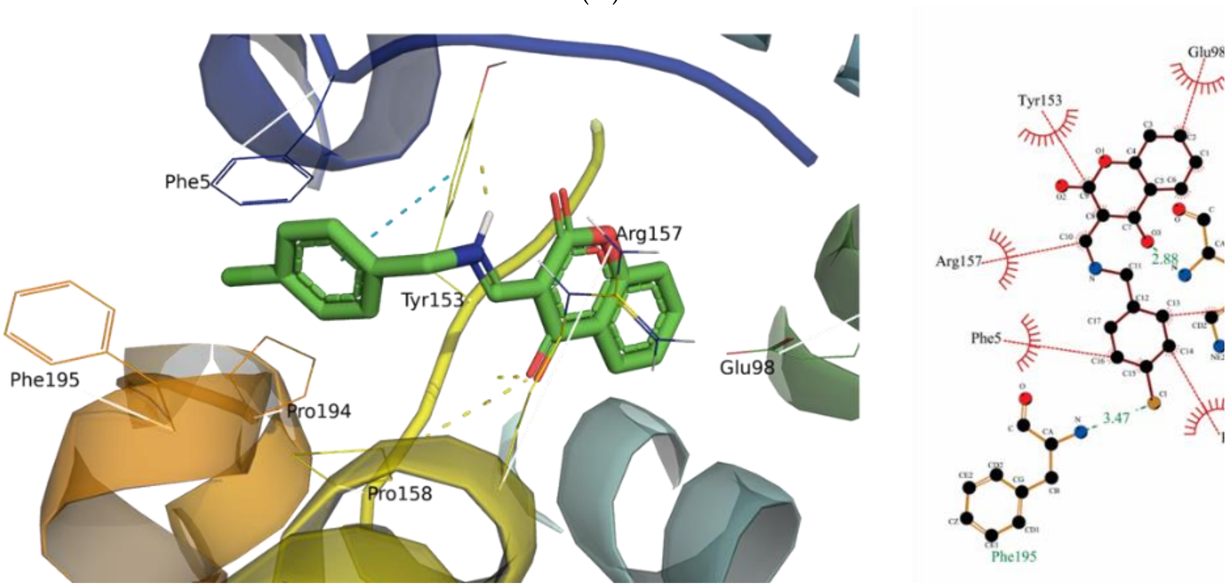

Phe5
Arg157
Tyr153
Glu98
Phe195
Pro194
Pro158
Glu98
Tyr153
Arg157
2.88
Phe5
3.47
Phe195


(4g)

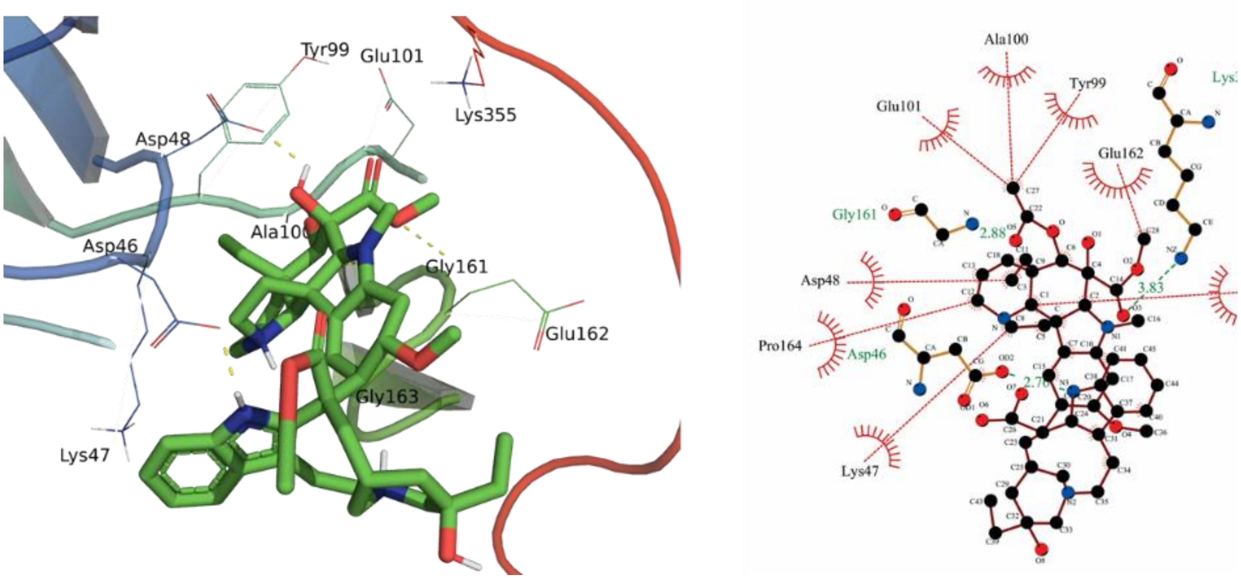


(4h) **Vinblastine**

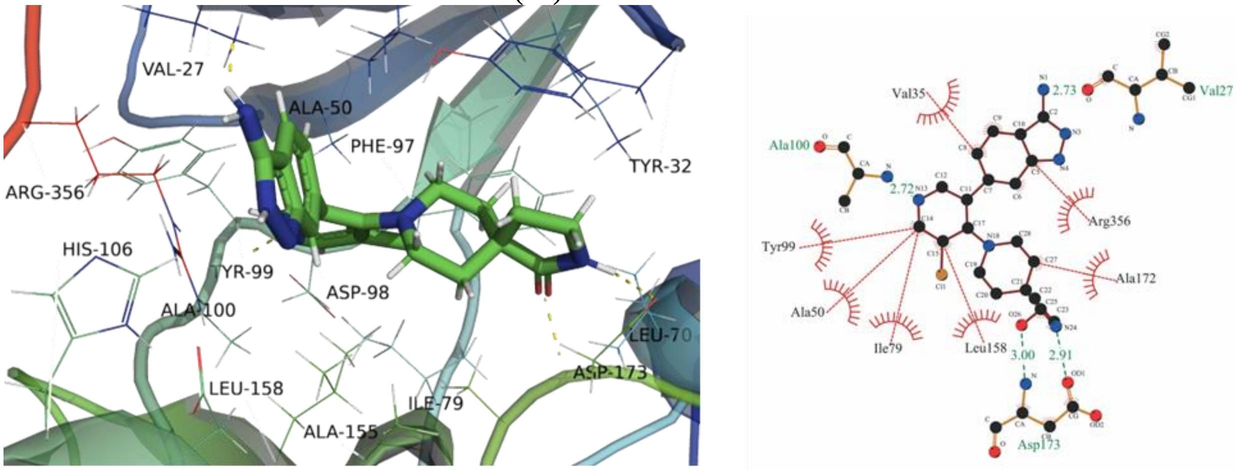


(4i) **Native ligand (5XG) of CDK-8**

**Figure S10.** Docking Simulations of Compounds (4a-i, Vinblastine and native ligand) with CDK-8 protein.
